# Metabolism: a potential regulator of neutrophil fate

**DOI:** 10.3389/fimmu.2024.1500676

**Published:** 2024-12-04

**Authors:** Zhou Yipeng, Cao Chao, Li Ranran, Pan Tingting, Qu Hongping

**Affiliations:** Department of Critical Care Medicine, Ruijin Hospital, Shanghai Jiao Tong University School of Medicine, Shanghai, China

**Keywords:** neutrophil, cell death, cell metabolism, glycolysis, apoptosis

## Abstract

Neutrophils are essential components of the innate immune system that defend against the invading pathogens, such as bacteria, viruses, and fungi, as well as having regulatory roles in various conditions, including tissue repair, cancer immunity, and inflammation modulation. The function of neutrophils is strongly related to their mode of cell death, as different types of cell death involve various cellular and molecular alterations. Apoptosis, a non-inflammatory and programmed type of cell death, is the most common in neutrophils, while other modes of cell death, including NETOsis, necrosis, necroptosis, autophagy, pyroptosis, and ferroptosis, have specific roles in neutrophil function regulation. Immunometabolism refers to energy and substance metabolism in immune cells, and profoundly influences immune cell fate and immune system function. Intercellular and intracellular signal transduction modulate neutrophil metabolism, which can, in turn, alter their activities by influencing various cell signaling pathways. In this review, we compile an extensive body of evidence demonstrating the role of neutrophil metabolism in their various forms of cell death. The review highlights the intricate metabolic characteristics of neutrophils and their interplay with various types of cell death.

## 1 Introduction

### 1.1 The life cycle of neutrophils

Neutrophils are essential innate immune system components, crucial for defending against pathogens including bacteria, virus and fungus (1). The homeostasis of neutrophils production and elimination is critical for appropriate immune system function. Neutrophils originate from multipotent hematopoietic stem cells located in the bone marrow or spleen. These stem cells undergo a series of maturation processes and eventually develop into mature neutrophils that are released into the bloodstream (2). Neutrophils production and maturation are regulated by various factors, including cytokines like CXCL8, growth factors like G-CSF, and cellular signals like PI3K/AKT pathway (3). Granulocyte-stimulating factor (G-CSF) is a key cytokine that enhances neutrophil production by promoting hematopoietic stem cell differentiation and proliferation (4). Mature neutrophils released into the bloodstream form the peripheral neutrophil reserve, which migrates to sites of infection or inflammation (5). On detection of invading pathogens or inflammatory signals, circulating neutrophils adhere to vascular endothelial cells and migrate into tissue spaces, guided by chemotactic factors, where they perform essential functions, such as bactericidal activity and phagocytosis (5).

Neutrophils generally have a relatively short lifespan, with half-lives ranging from several hours to a few days (6, 7). Timely elimination of aged, surplus, or non-functional neutrophils through cell death is crucial for maintenance of appropriate immune responses to various conditions (5, 8). Alteration of neutrophil cell death mechanisms can result in various pathological conditions, including acute inflammation, chronic inflammation and disruption of other immunological pathways (9–12). For example, delayed neutrophil death can exacerbate lung injury in patients with sepsis (13), while suppressed cell death within the microenvironment of certain solid tumors accelerates cancer metastasis (14). Therefore, understanding the detailed mechanisms underlying cell death is pivotal to full comprehension of a wide range of diseases.

Apoptosis, a programmed form of cell death commonly observed during inflammation, is the primary cell death mechanism that aids in clearance of unwanted neutrophils to prevent excessive inflammation (8, 15). However, various other types of neutrophil death have recently been detected, including autophagy, pyroptosis, necrosis, necroptosis, and newly-discovered mechanisms, such as ferroptosis (16–19). In addition to these conventional pathways, neutrophils undergo a specialized form of cell death, which is closely tied to their function, referredas NETosis (20, 21). These different cell death types have distinct roles in various physiological and pathological conditions, including inflammatory, non-inflammatory, pathogen-induced, and cell lysis-mediated contexts (22). It is crucial to determine the predominant mode of cell death occurring under specific conditions. Neutrophils that undergo programmed cell death are phagocytosed and cleared by surrounding macrophages, a process known as efferocytosis, which prevents secondary inflammation or autoimmune responses (23). Further, neutrophils undergoing other forms of cell death are cleared through dissolution and absorption, minimizing their potential harmful effects.

### 1.2 Neutrophilic function and disease

Neutrophils both function as first-line effectors in the innate immune response, defending against pathogens, such as bacteria, viruses, and fungi, and have roles in regulating various immunological processes, in contexts including cancer, injury, and tissue repair (24). Neutrophils can synthesize and release lysosomal enzymes, generate oxygen radicals, and release cytokines, underpinning their unique and crucial roles in the immune system (24–26). Due to their diverse functions and regulatory abilities, neutrophils have varying roles in the development of different diseases.

Neutrophils primarily function in protection against infections and tissue damage (24). As major components of the innate immune system, neutrophils eliminate pathogens through phagocytosis and by releasing toxic and antibacterial substances. Further, these cells can modulate immune responses by controlling the timing of pro- or anti-inflammatory cytokine release, thereby regulating inflammation intensity, to effectively remove infections without impairing tissue repair (26).

Abnormal neutrophil activity and numbers can have negative effects on disease development (24). Insufficient neutrophil activity increases susceptibility to opportunistic infections and allows infection spread. Conversely, excessive neutrophil infiltration can result in overwhelming inflammation, which delays tissue regeneration and increases the risk of reinfection (27). Further, dysregulated neutrophil activation can lead to overproduction of pro-inflammatory factors or insufficient anti-inflammatory factor generation, contributing to tissue damage in various organs and autoimmune diseases; for example, rheumatoid arthritis and Crohn’s disease (12, 24). Neutrophils are also crucial components of the tumor immunological microenvironment, where their functions and activities undergo specific modulation (28). In some cases, neutrophils aid tumor cell recognition and killing, while in others they promote tumor growth, metastasis, and drug resistance (28, 29). The mechanisms underlying the effects of neutrophils on tumors are complex and varied, and specific tumor cell types can exploit neutrophil immunoregulatory mechanisms to evade immune surveillance and accelerate tumor progression (30).

### 1.3 Immunometabolism and neutrophil death

Cell metabolism, which involves energy provision and biomolecule synthesis, is crucial for most cellular processes. Immunometabolism, referring to integration of metabolism in the immune system, has been a focus of immunology research for some years. Both intercellular and intracellular metabolic signaling strongly influence the activation state and fate decisions of immune cells, including neutrophils. Neutrophils can rapidly transition from quiescent to activated states, which requires dynamic metabolism to meet their energy and biosynthetic demands. Metabolic signaling operates bidirectionally, in that cellular signaling can reprogram metabolism, while metabolites play critical roles in multiple signaling pathways. Specific cellular processes trigger the activation of distinct metabolic pathways, enabling cell adaption, underscoring the critical role of metabolic fluctuations (31). Neutrophils have traditionally been considered glycolysis-dependent cells that primarily derive energy from glucose (32, 33). However, recent studies have highlighted the importance of other metabolic pathways, including mitochondrial, lipid, and glutamine metabolism, in the neutrophil lifespan and their functions (34–37).

Few studies to date have linked neutrophilic metabolism with cell death, and the comprehensive metabolic features associated with specific types of cell death remain unclear. Reactive oxygen species (ROS) have pivotal roles in various forms of cell death (38–40). utilize multiple metabolic pathways to generate ROS, which can be synthesized in the cytoplasm or mitochondria (17, 34, 35, 41). Nevertheless, the precise mechanisms governing ROS production and utilization in specific types of neutrophil cell death remain incompletely elucidated, and warrant further research. Furthermore, the processes of neutrophil death can be influenced by metabolites other than ROS (41, **Table 1**).

**Table 1:** ROS production in different processes in neutrophils.

Differentiation	Activation	Apoptosis	NETosis
Production↑ (44, 45)	Production↑ (44, 45)	Production ↑(early) (107) Production ↓(late) (110, 111)	Production ↑ (132)
Necrosis/necropotosis	autophagy	pyroptosis	ferroptosis
Production ↑ (155)	Production ↑ (165)	Production ↑ (176, 177)	Production ↑ (193, 194)

Arrow ↑ Means ROS production is promoted in the cellular process, Arrow ↓ means ROS production is inhibited in the cellular process.

In this review, we provide an overview of the fundamental metabolic profiles of neutrophils in both quiescent and activated phases. Additionally, we summarize the known relationships among neutrophil metabolic reprogramming and various cell death processes. Our aim is to outline a concise map of metabolic transitions throughout the lifespan of neutrophils and offer insights that can inspire future research (**Table 2**).

**Table 2:** Metabolism alteration in different processes in neutrophils.

Cell process	Glucose metabolism	Glutamine metabolism	Lipid metabolism
**Differentiation**	Glycolysis↑ (49) OXPHOS blocked (49)	Unknown	Lipolysis ↑ (78)
**Activation**	Glycolysis↑ (49) PPP↑ (59) Glycogenolysis ↑ (52)	Glutaminolysis ↑ (37, 95)	Lipid metabolism inhibited (78)
Cell death	Glucose metabolism	Glutamine metabolism	Lipid metabolism
**Apoptosis**	Glycolysis↓ (110, 111) PPP↓ (110, 111)	Glutaminolysis ↓ (37)	Phospholipolysis ↑ (122)
**NETosis**	Glycolysis↑ (136, 137) PPP↑ (141)	Glutaminolysis ↑ (138) Take-up ↑ (138)	under pathological conditions↑ (142, 144, 147, 150)
**Necrosis/necroptosis**	Unknown	Unknown	Unknown
**Autophagy**	Glycolysis↓ (163)	Unknown	Lipolysis ↑ (78)
**Pyroptosis**	Glycolysis↑ (178, 179)	Unknown	FAO↑ (180) Ketone body production↑ (182)
**Ferroptosis**	Take-up↑ (196)	Glutaminolysis ↑ (193–195) Take-up ↑ (193–195)	FAO↑ (187) Phospholipid oxidation ↑ (187)

Bold texts indicate different cellular processes. Arrow ↑ Means the metabolic activity is promoted in the cellular process, Arrow ↓ means the metabolic activity is inhibited in the cellular process.

## 2 Fundamental metabolism profile of neutrophils

In this section, we discuss the primary metabolic pathways in neutrophils. Although neutrophils rely on glycolysis to provide most of the energy required for their basic functions (32, 33), different types of metabolism are activated in neutrophils during phagocytosis and other immunological processes (34, 42, 43). Oxygen consumption and ROS production are remarkably promoted as neutrophils transition into an active state, and this phenomenon is referred to as respiratory burst (44, 45). Glycolysis and other neutrophilic metabolic processes are influenced by respiratory burst, despite glycolysis generally being considered an anaerobic pathway (46). Other metabolic alterations also warrant deep investigation, to facilitate comprehensive understanding of neutrophil metabolism.

### 2.1 Glucose metabolism

Glucose is the major substance used to supply energy in neutrophils (34). Neutrophils obtained from the surrounding environment via the glucose transport proteins, GLUT1, GLUT3, and GLUT4, which are expressed on neutrophil cytomembranes (47, 48). When neutrophils are in a quiescent state, GLUT1 serves as the chief glucose transporter; however, on activation, there is a marked increase in GLUT3 and GLUT4 expression, which ensures sufficient glucose supply (48).

**Figure 1:**
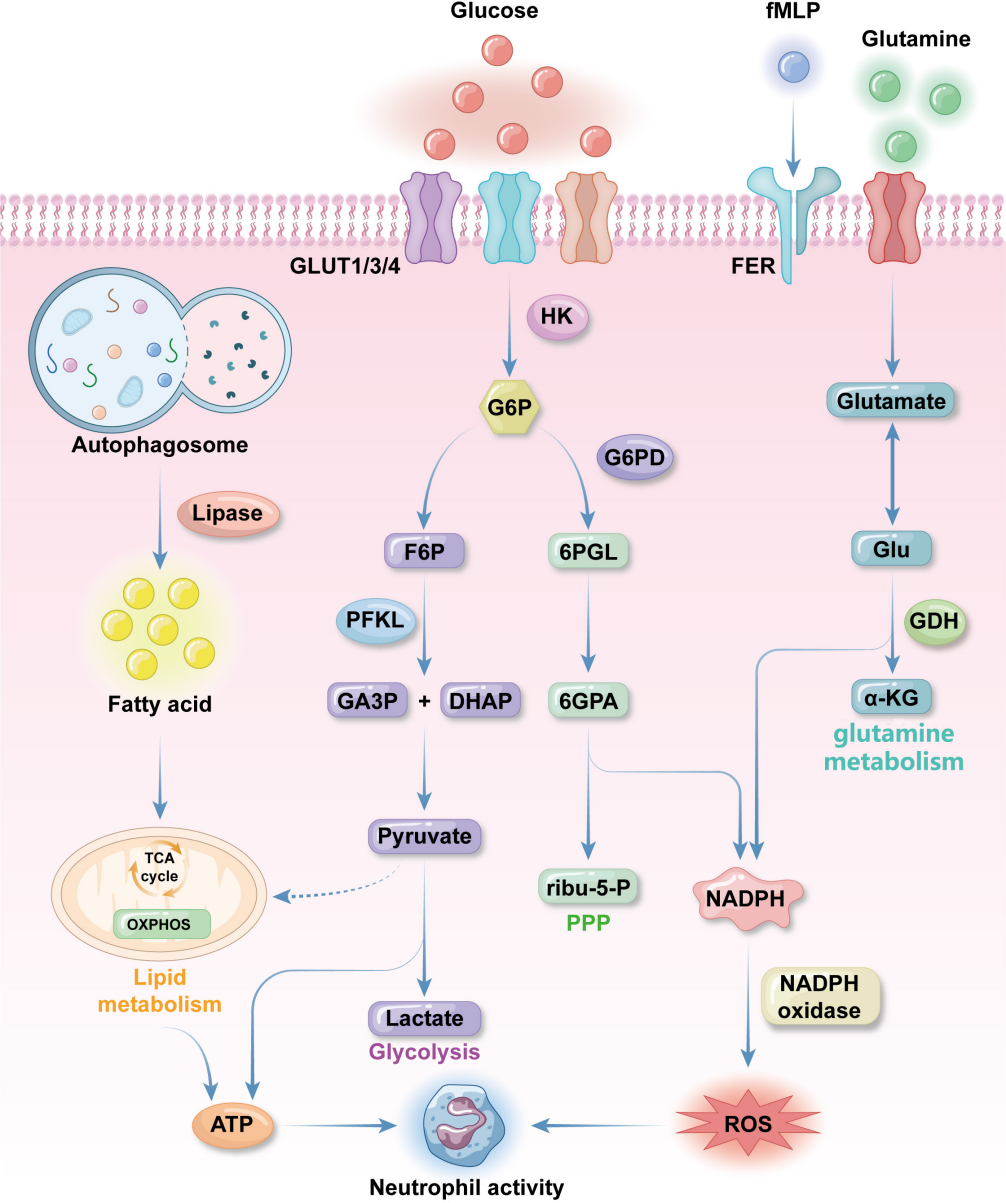
Fundamental metabolic features of neutrophils. neutrophils uptake glucose by the effect of glucose transporter proteins GLUT1, GLUT3 and GLUT4 expressed on the plasma membrane. Glucose will be converted to glucose-6-phosphate (G6P) by hexokinase 2(HK2) as immediately as glucose enter neutrophilic cytosol. G6P will be turned into 3-phosphoglyceraldehyde(GA3P) and dihydroxyacetone phosphate(DHAP), pyruvate and lactate in sequence for ATP synthesis in glycolysis. When neutrophils are activated pentose phosphate pathway (PPP) will be activated. PPP get 6-phosphogluconolactone(6PGL) from G6P through G6P dehydrogenase and finally get NADPH, which produce reactive oxygen species (ROS) for anti-bacterial activities. Glutamine is an important supplementation source of ROS. It is turned into glutamate, which can be transformed into Glu and then α-KG to get NADPH. Glutamine uptake and utilization can be promoted by formyl peptide receptors (FPR) with N-formyl-L-methionyl-L-leucyl-phenylalanine (fMLP) to promote NADPH synthesis. Lipid metabolism is the major energy source during neutrophils differentiation. Autophagosome use lipase to degrade lipid into fatty acids (FAs), which then get into TCA cycle to produce energy for differentiation and other neutrophils activities. Conventional mitochondrial metabolism pathways like TCA cycle and oxidative phosphorylation (OXPHOS) only occur before neutrophils finish differentiation because mature neutrophils contain a low density of mitochondria with low activities.

As progenitor myeloid cells differentiate into neutrophils, their main energy source shifts from oxidative phosphorylation (OXPHOS), which connects the tricarboxylic acid (TCA) cycle with the electron transport train in aerobic respiration, to glycolysis (49). This transition allows neutrophils to function effectively in inflammatory environments, which are typically low in available oxygen, and is triggered by hypoxia inducible factor-1α (HIF-1α). Hypoxia inhibit the hydroxylation of H1F-1α by prolyl hydroxylase domain family and factor inhibiting HIF (FIH) dioxygenases to permit function of HIF-1α. HIF-1α is a transcriptional factor that promote the expression of glucose transporters like GLUT1 and GLUT3 and glycolytic enzymes like aldolase and phosphoglycerate kinase-1 (50). HIF-1α can also suppress OXPHOS byenhancing the expression of pyruvate dehydrogenase kinase 1and and LDHA to prevent pyruvate from being turned into actyl-CoA that enter TCA cycle. Once glucose enters neutrophils, it is immediately converted into glucose-6-phosphate (G6P), to prevent it leaving the cell. Under aerobic conditions, G6P is transformed into lactate, the ultimate product of glycolysis, rather than remaining as pyruvate for entry into the TCA cycle (33, 51) (**Figure 1**).

In some contexts, for example inflammatory sites or tumor microenvironments, there is limited glucose available to neutrophils; therefore, to maintain glucose homeostasis, neutrophils also have robust capacity to store and utilize glycogen (33). Glycogen storage dysfunction or replenishment leads to metabolic exhaustion and impaired neutrophil function, resulting in deficient neutrophil-related immune responses (52). G6P, which bridges glycolysis and glycogenesis, is a key regulator of glucose and glycogen homeostasis (53). The glucose-6-phosphotase (G6Pase) complex, which localizes to the endoplasmic reticulum membrane, converts G6P to glucose, to maintain glucose and glycogen balance, rendering neutrophils the only leucocytes equipped with both gluconeogenesis and glycogenesis abilities (54). Glyceraldehyde-3-phosphate, an intermediate product of glycolysis, also regulates this process (55). Impaired glycogen synthesis from G6P leads to the autosomal-recessive syndrome, glycogen storage disease type Ib (GSD-1b), characterized by neutropenia and neutrophil dysfunction (56). GSD-1b is caused by deficiency in glucose-6-phosphate transporter (G6PT), as G6Pase-β function is closely associated with G6PT (57). The neutropenia observed in GSD-1b results from over-activated neutrophil apoptosis and abnormal neutrophil differentiation in the bone marrow, influenced by disordered energy supplementation and activation of transcription factors, including HIF-1α and PPAR-γ (58).

As mentioned above, activated neutrophils require ROS for their immunological functions. A major route to ROS production in activated neutrophils is through the pentose phosphate pathway (PPP) in the cytosol (59). Although glycolysis does not directly produce ROS, it does generate G6P for the PPP (60). Unlike the TCA cycle, the PPP utilizes G6P to efficiently synthesize large amounts of NADPH for ROS production, without generating any ATP (61, 62). Once neutrophils are stimulated, the PPP will be strongly triggered within 30 minutes in preparation for an immune response (63). G6P dehydrogenase is an important enzyme that catalyzes the conversion of G6P to 6-phosphogluconolactone, which initiates the PPP (61). G6PD deficiency is a genetic disorder characterized by susceptibility to infections and recurrent bacterial infections, due to insufficient NADPH and ROS production (64). Cytoplasmic ROS derived from the PPP is crucial in mediating the release of neutrophil extracellular traps (NETs), a process linked to both neutrophil immune effects and cell death (65) (**Figure 1**).

### 2.2 Mitochondria metabolism

In the quiescent state, neutrophils have low mitochondria density and weak mitochondrial activity, and the role of mitochondria in neutrophil function remains controversial (66, 67). Mitochondrial respiratory chain inhibitors, such as FCCP or oligomycin, which deplete mitochondrial membrane potential and depress mitochondrial ATP synthesis, respectively, do not decrease ATP production or basal oxygen consumption in neutrophils (68, 69). Further, temporary treatment using mitochondrial respiratory chain inhibitors has no influence on ROS production; however, prolonged mitochondrial suppression using oligomycin (incubation > 2 h) results in reduced ability to trigger respiratory burst (69). A metabolic shift from OXPHOS to glycolysis occurs during neutrophil differentiation in the bone marrow, and OXPHOS is a primary source of mitochondrial ROS (mROS) in many cell types, distinct from the cytosolic ROS synthesized by membrane receptors, such as formyl peptide receptors (FPR), during respiratory burst (70, 71). Bao et al. demonstrated that N-Formyl peptides expressed on invading bacteria (e.g., N-formyl-L-methionyl-L-leucyl-phenylalanine, fMLP) can stimulate neutrophilic respiratory burst by activating FPRs, and this interaction can be blocked by oligomycin (71). Subsequent studies clarified that ROS induced by fMLP originates from the cytosol and does not involve mROS production (72). It indicates that mitochondria may sense neutrophilic respiratory bursts, independently of OXPHOS (73) (**Figure 1**).

Mitochondria loss is also driven by the effects of HIF-1α on transcription during differentiation. Hypoxia activate HIF-1α to induce mitochondrial autophagy in neutrophils (50). Cytochrome c, a vital component of the electron transport chain, is downregulated on HIF-1α activation, leading to OXPHOS suppression (49). Another essential function of OXPHOS is creating membrane electrochemical potential (ΔΨm) across the mitochondrial membrane, which facilitates electron transfer and provides energy for ATP synthesis (74). Electron transfer is mediated by four mitochondrial respiratory chain complexes (CI, CII, CIII and CIV) in the inner mitochondrial membrane (75). The absence of cytochrome c results in loss of CI, CIII, and CIV within a respiratory chain, with consequent failure to produce sufficient energy for ATP synthesis (51, 75, 76). Although ΔΨm does not typically contribute to neutrophil energy supplementation and biosynthesis, diminished mitochondrial membrane potential can trigger neutrophil apoptosis (49, 69). To maintain cell viability, neutrophils utilize glycerol-3-phosphate to transport electrons. The ΔΨm generated with glycerol phosphate as a substrate is higher than that generated with other complexes, with or without substrate. Glycerol-3-phosphate can diffuse into the mitochondria immediately on its production during glycolysis, followed by oxidation to dihydroxyacetone-phosphate on the outer surface of the inner mitochondrial membrane. This process donates electrons to complex III of the respiratory chain via ubiquinol (46). The mROS inhibitor, MitoTEMPO, accumulates in mitochondria and reduces neutrophil superoxide production by inhibiting complex III, highlighting the critical role of complex III in mROS production (77). Inhibiting complex III also increases lactate production, as it prevents glycerol-3-phosphate from entering mitochondria. This process is considered to regulate aerobic glycolysis, without affecting ATP synthesis (46) (**Figure 1**).

Fatty acid oxidation (FAO) is another important feature of neutrophil mitochondrial metabolism in ATP-demanding situations, such as differentiation or tumor adaptation (78, 79). Concerning the unique role of lipid metabolism in neutrophil, it will be discussed in the next part.

### 2.3 Lipid metabolism

In recent years, there has been an increasing focus on lipid metabolism as an additional energy source, alongside glycolysis, in various neutrophil functions (36). Lipid metabolism serves as an important contributor to energy provision, cell signal transduction, and immune response regulation, under both physiological and pathological conditions.

Fatty acids (FAs) are essential energy sources for neutrophils, particularly when glucose is limited (79). Autophagy is the main producer of FAs in neutrophils through lipophagy, which supplies the majority of energy for neutrophil differentiation (78).During differentiation, lipid can be degraded into FAs, which is then converted into TCA cycles to synthesize ATP and provide most energy for cellular processes of differentiating neutrophils. PPARγ is a transcription factor that regulate adipogenesis and promote lipid droplet formation (80). G-CSF can stimulate the accumulation of LDs in neutrophils and accelerate the maturation of neutrophils through PPARγ (81).During infection, Platelet activating factor, LPS stimulation and cytokines like IL-5 facilitate LDs accumulation with signal platform of TLR4, TLR7 and TLR9 to enhance the immune response (82, 83). Lipophagy involves the transport of lipid droplets, which are membrane-bound lipid-storing particles in the cytosol, into double membrane-bound autolipophagosome vesicles, which are then delivered to lysosomes for oxidation into FAs (84). These FAs are then converted into fatty acyl-CoA esters, which are transported into mitochondria via an acetylcarnitine transporter. Once inside the mitochondria, fatty acyl-CoA esters are metabolized into acetyl-CoA molecules, which enter the TCA cycle and the OXPHOS system to generate ATP and NADPH (85). Neutrophil maturation and differentiation are predominantly regulated by autophagy related protein 7 (ATG7) in mice, deficiency of which disrupts lipophagy, hindering neutrophil differentiation and causing accumulation of immature neutrophils in the bone marrow (78). Besides autophagy, FAs can also be derived from the decomposition of excess lipids by neutral lipases, such as adipose triglyceride lipase and hormone-sensitive lipase, which help cover energy shortages under metabolic stress (86, 87) (**Figure 1**).

In inflammatory pathways, lipids serve as both independent signaling molecules in cell signal transduction, and as activators or modulators of certain effector proteins or transcription factors (88, 89). Lipid inflammatory mediators can be classified into three types according to their structures: arachidonic acid-derived eicosanoids, membrane phospholipids, and omega-3/6 FA-derived lipids (36). These molecules are vital regulators of proper immune responses, as myeloid-specific adipose triglyceride lipase-deficient mice, which fail to synthesize lipid mediators, exhibit disordered immunological features (90). Fatty acid transporters and fatty acid recognition receptors on neutrophils can also efficiently modulate inflammatory pathways by interacting with specific lipids in the immunological microenvironment to enhance innate immune responses, or promoting neutrophil recruitment to inflammatory sites (91, 92).

### 2.4 Glutamine metabolism

Glutamine is an amino acid which remarkably modulates immune function in the human body. Neutrophils are among the immune cells that utilize glutamine and consume it at a higher rate than other leukocytes (93) (94). Anti-infection function of neutrophils can be facilitated by glutamine without influencing their phagocytic ability (95). This effect of glutamine on neutrophil function likely mediated by increased ROS production, as glutamine can generate NADPH through malate synthesis and activated NADPH oxidase, a key enzyme in ROS production, by upregulating the expression of its components, gp91, p22, and p47 (96) (**Figure 1**).

Deficient glutamine utilization or inadequate glutamine supply is strongly associated with various diseases, including cancers, diabetes, and sepsis, which may result from oxidative stress (97–99). Glutathione/γ-l-glutamyl-l-cysteinyl-glycine (GSH), the most abundant non-enzymatic antioxidant in human cells, is primarily distributed in the cytoplasm, and can directly react with ROS to eliminate peroxides (100). Oxidized GSH (GSSG) is produced during this reaction, thus cell redox state can be reflected by the GSH/GSSG ratio (101). Glutamine, in the form of glutamate, is a crucial precursor amino acid for GSH and has a vital role in GSH synthesis; hence, glutamine levels determine the GSH/GSSG ratio by modulating GSH synthesis, which facilitates neutrophil function under oxidative stress (98).

## 3 Metabolic alterations in different types of neutrophil death

### 3.1 Apoptosis

Apoptosis is among the most common forms of cell death in neutrophils under physiological conditions. Neutrophil apoptosis usually results from caspase activation through various pathways, and is characterized by changes in cell shape, pseudopod retraction, volume reduction, and chromatin condensation (102–104), and observation of these specialized changes under a microscope indicate that apoptosis is occurring.

Neutrophil apoptosis initiation can be divided into two main pathways, intrinsic and extrinsic. The extrinsic pathway involves activation of death receptors and members of the tumor necrosis factor receptor family, distributed on the plasma membrane (105, 106). Death receptor pathways, such as CD95 (Fas/APO-1), can respond to exogenous and endogenous ROS in neutrophils, inducing apoptosis (107). The intrinsic pathway is triggered by cytochrome c release from mitochondria into the cytosol, regulated by the bcl-2 family of apoptotic proteins, which maintain mitochondrial integrity (108). Cytochrome c interacts with the cytosolic protein, apoptotic protease-activating factor 1 (Apaf-1), to activate the effector, caspase-9 (109). Further, cytochrome c is released as the outer mitochondrial membrane is destroyed, due to loss of mitochondrial membrane potential, which can be diminished by treatment with mitochondrial respiration inhibitors, such as oligomycin, to initiate apoptosis even before apoptotic morphological features appear (69). Nevertheless, inhibiting mitochondrial function does not accelerate neutrophil apoptosis (69).

**Figure 2:**
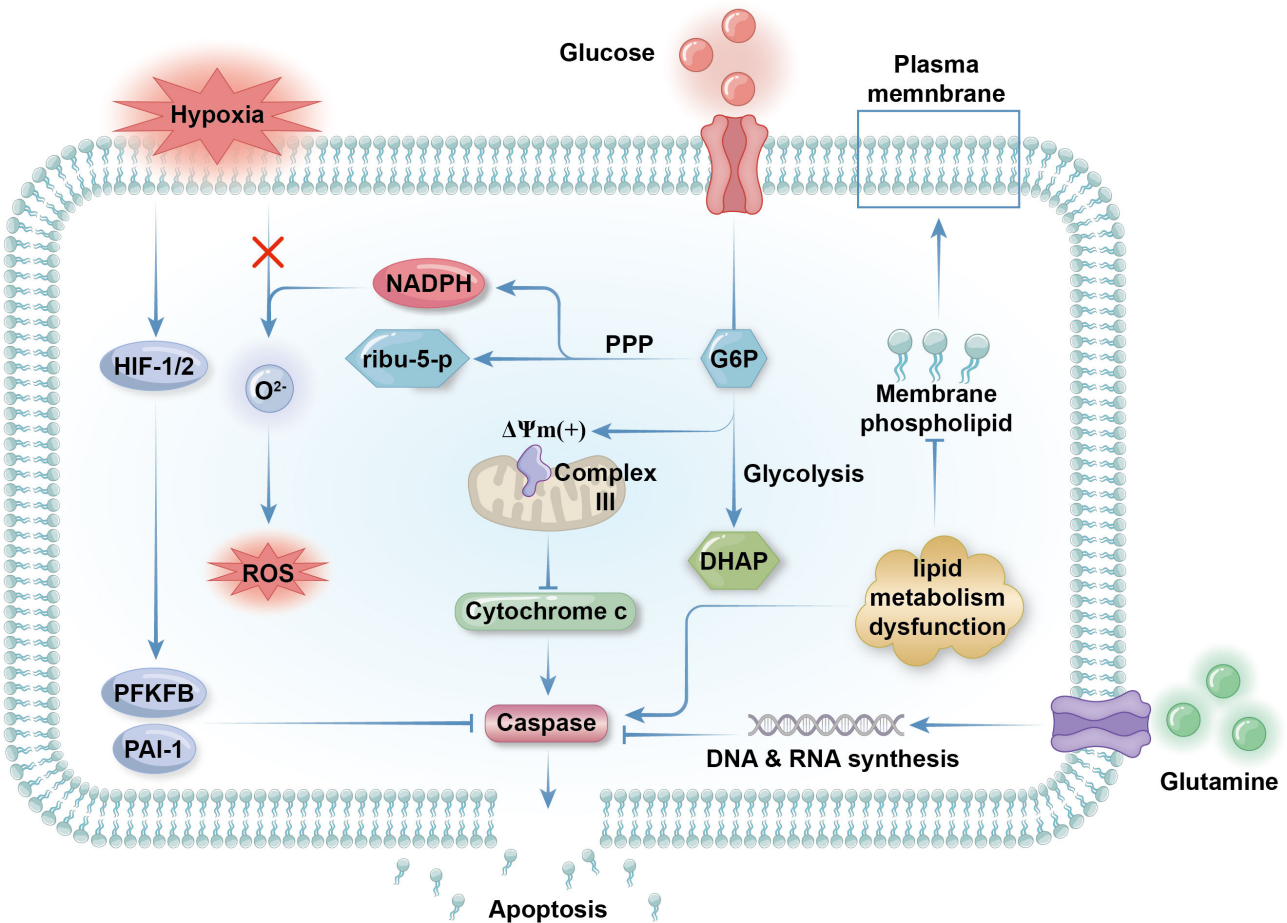
Metabolic regulation of apoptosis. Glycolysis is inhibited as neutrophilic apoptosis is triggered. Glycolysis contributes membrane electrochemical potential (DYm) to respiratory chain complex III expressed on the outer membrane of mitochondria to maintain the integrity of the organelles. If glycolysis is low, the mitochondria will rupture and release cytochrome c into the cytosol, which activates apoptotic effector caspase and initiate the programmed cell death. Hypoxia delay apoptosis by activate transcriptional factors HIF1 and HIF2 as the promote the expression of anti-apoptotic protein like PAI-1 and 6-phosphofructo-2-kinase (PFKFB). Concerning that ROS can initiate apoptosis by activating death receptors like CD95 on plasma membrane, preventing ROS production by limiting peroxide like O_2_^2-^ is another way of hypoxia rejecting apoptosis. Although lipid metabolism is in a low state in mature neutrophils, impaired lipid metabolism that inhibit the synthesis of membrane phospholipid can result in apoptosis as well. Glutamine metabolism get weaker during apoptosis because of decreased demand for NADPH in apoptotic neutrophils. Surviving cells need abundant glutamine supply for the synthesis of nucleic acid for the transcription and expression of anti-apoptotic genes.

Glycolysis is inhibited in apoptotic neutrophils, which cannot trigger respiratory bursts, leading to decreased pathogen killing ability (110, 111). As previously mentioned, mitochondrial membrane maintenance in neutrophils is fueled by glycerol-3-phosphate produced during glycolysis, suggesting that reduced glycolysis may trigger neutrophil apoptosis (46). Limited glucose (0.6 mM) is a key promoter of neutrophil apoptosis, an effect enhanced by G6Pase-β deficiency in patients with GSD-Ib (112). G-CSF, which is used to treat neutropenia in various conditions, can delay, but not prevent, neutrophil apoptosis by repairing glucose uptake and utilization (112). Unlike in other cells, hypoxia delays apoptosis in neutrophils, both by reducing the ROS levels crucial for apoptosis, and by activating HIF family transcriptional factors (107, 113–115). HIF-1α primarily modulates neutrophil metabolism for adaptation to anoxic environments (114). Li et al. demonstrated that cyclosporine can inhibit the SIRT6-HIF-1α-glycolysis axis to accelerate neutrophil apoptosis (116). In contrast, HIF-2α activity directly promotes the intrinsic apoptosis pathway (115). These mechanisms help prolong the lifespan of neutrophils and reinforce immune function in low-oxygen sites of inflammation (117) (**Figure 2**).

Glutamine metabolism is crucial for NADPH generation and is downregulated in apoptotic neutrophils, due to reduced NADPH demand. Besides boosting respiratory bursts, glutamine also produces precursor nucleotides for RNA and DNA synthesis, and is responsible for expression of surface activation proteins and cytokine production (118). Glutamine supplementation can effectively slow neutrophil apoptosis, particularly exercise-induced apoptosis, and modulates immunological functions (37, 119). While glutamine fuels ROS generation, it suppresses extrinsic apoptosis by decreasing p38, MAPK, and JNK phosphorylation and reducing p53 and caspase-3 expression (120). Interestingly, glutamine extends the life span of neutrophils and enhances their function while exhibiting anti-inflammatory effects, possibly due to its protective effects on other cells (121) (**Figure 2**).

The effects of lipid metabolism regulation on neutrophil lifespan are variable. Neutrophils barely utilize FAO at the end of their lives, when their energy needs are markedly reduced. Dysfunction of lipid metabolism can suppress peroxisome-derived membrane phospholipid synthesis, leading to neutrophil apoptosis without affecting neutrophil energy metabolism or differentiation (122). Inflammatory mediators derived from lipolysis are important in propelling neutrophil apoptosis, mainly through death receptor signaling pathways (102). For example, 15-deoxy (Delta)12-14PGJ2, a cyclopentenone prostaglandin derived from arachidonic acid, induces neutrophil apoptosis in rats, possibly through PPARγ inactivation (123, 124). Phospholipolysis is a vital source of arachidonic acid in neutrophils, and phospholipase and sphingomyelinase activity, which produce endogenous lipid mediators from membrane phospholipids, are closely associated with normal apoptosis progression (125). Overall, lipid metabolism can have dual effects on neutrophil survival, and the purpose of lipolysis and lipogenesis in neutrophils varies considerably in the contexts of different diseases (**Figure 2**).

Neutrophil apoptosis can be caused by extracellular stimulation or damage, as well as by scarcity of anti-apoptotic molecules, including anti-apoptotic proteins and pro-inflammatory signaling molecules (36, 126). Anti-apoptotic proteins, for example, the bcl-2 family member, Mcl-2, are highly expressed in neutrophil cytosol and their levels decrease before other apoptotic changes occur (126). As Mcl-2 has a shorter half-life than neutrophils (around 2–3 h) and neutrophils do not express other bcl-2 family proteins, vigorous protein synthesis is required for neutrophil survival, and impaired protein and amino acid metabolism can lead to apoptosis (**Figure 2**).

NETosis is a specialized form of cell death in neutrophils that shares many mechanistic features with apoptosis. NETosis and apoptosis appear to be two sides of the same coin, with various factors determining which pathway a dying cell will take. The mechanisms, similarities, and differences between NETosis and apoptosis are discussed in the following section.

### 3.2 NETosis

NETs are web-like chromatin structures released by neutrophils that are important for neutrophil immune responses and pathogen elimination (65). Accompanied by nucleus disintegration, cell membrane collapse and cell dysfunction, NET formation and release are features of a unique type of cell death, differing from apoptosis and necrosis, NETosis (127, 128). However, in some cases, neutrophils can maintain biological and immunological function after releasing NETs (129). Hence, NETosis is categorized according to its effect on neutrophil viability as suicidal and vital NETosis (129). Although suicidal and vital NETosis share notable common mechanisms among their key steps, including NET assembly and release, they lead to vastly different outcomes for neutrophils. Therefore, the Cell Death Nomenclature Committee does not recommend using the term NETosis to describe the vital process of NET release (130). In this review, we focus solely on the metabolic mechanisms that are either shared between vital and suicidal NETosis or exclusive to suicidal NETosis.

**Figure 3:**
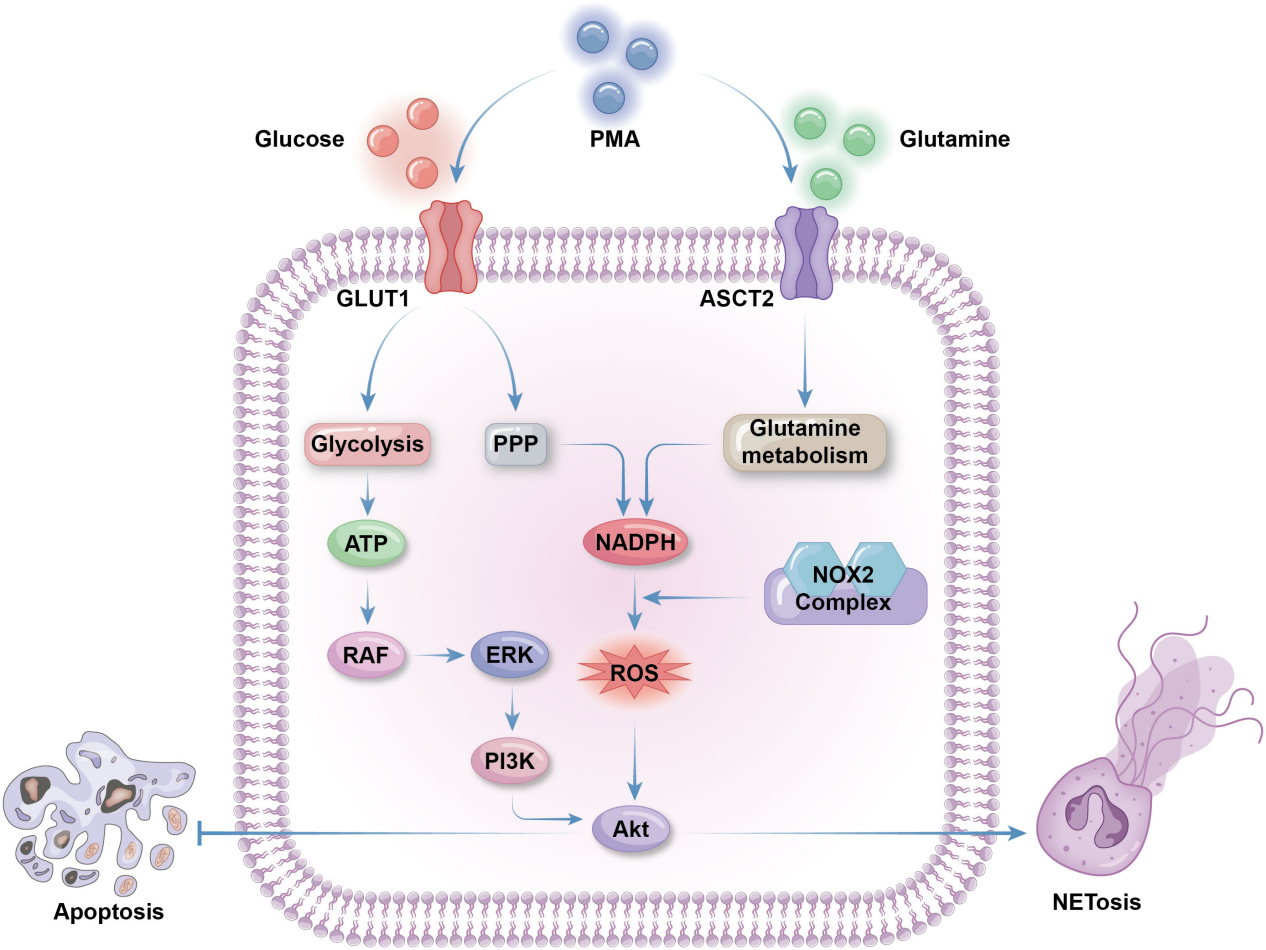
Metabolic selection of apoptosis or NETosis. The initiation of NETosis greatly depends on glucose metabolism, including glycolysis to produce ATP and PPP to produce NADPH. Glycolysis activate RAF via ATP production. RAF then activate ERK to trigger PI3K/AKT pathway to promote NETosis and inhibit apoptosis. Cytosolic ROS produced by NADPH oxidases 2(NOX2) is necessary for AKT production. Besides of enhanced PPP activity, glutamine metabolism is also facilitated to supply extra NAPDH for NETosis. Both glucose and glutamine uptake and metabolism can be enhanced by NETosis activator phorbol 12-myristate 13-acetate (PMA) by promoting the expression of membrane glucose transporter GLUT1 and membrane glutamine transporter ASCT2.

Neutrophil activation is required to trigger NETosis; hence, the metabolic features of NETosis initiation are aligned with those of neutrophil activation, including upregulation of glycolysis, lipid metabolism, and respiratory burst (34, 131). Neutrophils can produce ROS through NADPH oxidase activity and utilize it for NETosis (132). NETosis and apoptosis are generally considered mutually exclusive cell death processes that contribute to opposing metabolic alterations. Although ROS can activate both NETosis and apoptosis, the Akt and JNK pathways mediate the switch from apoptosis to NETosis in a ROS production-dependent manner (133, 134). Phorbol 12-myristate 13-acetate (PMA), a widely used NETosis activator, enhances the activity of NADPH oxidase 2 (NOX2), leading to synthesis of large amounts of cytosolic ROS, thus triggering Akt pathway to promote NETosis and inhibit apoptosis (134). Akt can also be activated to initiate NET release, as sensor kinases in the JNK pathway are triggered on stimulation by gram-negative bacteria or high levels of lipopolysaccharide (133). Meanwhile, recent evidence indicates that, in some specific cases, such as ultraviolet stimulation, another type of cell death, aponetosis, with features of both NETosis and apoptosis, is triggered through an NADPH oxidase-independent pathway; the metabolic features of aponetosis require further investigation (135) (**Figure 3**).

Abundant glucose is indispensable for NETosis, and glycolysis is the main glucose metabolic pathway that fuels NETosis. In the context of diseases associated with overactivated NETosis, such as cystic fibrosis or asthma, neutrophils from airway connective tissue contain increased levels of glycolytic metabolites and exhibit enhanced glucose uptake (136, 137). 2-deoxy-D-glucose, a glucose analogue that competitively binds to the glycolytic rate-limiting enzyme, glucose hexokinase, blocks NETosis by inhibiting glycolysis (138). NET formation can be divided into two phases: the first phase is chromatin decondensation, which does not rely on glycolysis; while the second is glycolysis-dependent NET release. Activated neutrophils grown on glucose-free media undergo morphological changes to their nuclei, without release of NET vesicles (138). PMA-stimulated NETosis and NETosis in cystic fibrosis are accompanied by upregulated expression of GLUT1, rather than GLUT3 or GLUT4, which is a primary contributor to enhanced glucose metabolism (138, 139). As discussed above, PPP is the most important source of ROS production in neutrophils, producing ample NADPH for NADPH oxidase activity. The downstream glycolytic enzyme, phosphofructokinase liver type (PFKL), converts fructose-6-phosphate (F6P) to fructose-1,6-bisphosphate, preventing F6P entry into the PPP, thus blocking NADPH and ROS production (140). Hence, proper PPP initiation is necessary for ROS supply for NETosis, despite enhanced glycolysis (141) (**Figure 3**).

There is a weak association between lipid metabolism and NETosis levels under physiological conditions, as NETosis usually occurs in mature neutrophils with little reliance on lipid metabolism for energy production (102). However, in some diseases, NETosis can be abnormally activated through certain specialized lipid metabolism pathways. LNK is a member of the SH2B family of adaptor proteins that acts as a key regulator of hematopoietic cell signaling and proliferation (142, 143). Together, LNK deficiency and hyperlipidemia lead to platelet production and activation, platelet–neutrophil aggregates, and neutropenia caused by overwhelming NETosis, resulting in vascular or coagulation disorders (144, 145). LNK dysfunction promotes neutrophil priming and response to oxidized phospholipids from activated platelets, accelerating NETosis. This process can be completely blocked by deficiency of peptidyl arginine deiminase 4 (PAD4), a key effector of DNA decondensation (145). Disruption of wound healing and cardiovascular disorder in patients with diabetes or model mice may also be a consequence of NETosis mediated by abnormal lipid metabolism and PAD4 function (146, 147). Insulin resistance promotes fatty acid uptake and oxidation, leading to increased ROS production in neutrophils. Excessive ROS stimulates the function of transcription factors, such as PPARα, and the expression of enzymes, including PAD4, which help to trigger NETosis (148, 149). During the development of atherogenesis, cholesterol facilitates neutrophil recruitment and NET formation, which is mediated by activation of the proinflammatory NOD-like receptor family, pyrin domain-containing 3 (NLRP3) inflammasome protein (150). Unusual lipid metabolism in various cells, such as cholesterol accumulation in macrophages within atherosclerotic plaques, can also stimulate NETosis, due to excessive secretion of IL-1β by these macrophages (151) (**Figure 3**).

Glutamine is crucial for NADPH oxidase activity and ROS production, making it significant for NETosis initiation. PMA stimulates expression of the NADPH oxidase components, gp91, p22, and p47, to facilitate NET release, which can be blocked by the glutamine metabolism inhibitor, 6-Diazo-5-oxo-l-norleucine (96). Glutamine depletion partially, but not completely, diminishes NET formation, as glucose is the primary source of ROS for most neutrophilic antibacterial activities (138). Macrophages use glutamine to synthesize nitric oxide (NO) from arginine through the inducible NO synthase enzyme, utilizing NADPH as an energy source; however, there is minimal evidence supporting this pathway in neutrophils (**Figure 3**).

### 3.3 Necrosis and necroptosis

Necrosis is a form of cell death that is less regulated than apoptosis, which is initiated in response to detrimental environmental factors, such as lack of oxygen or essential nutrients, toxic substances, or extremely acidic or basic conditions (103). Similar to apoptosis, necrosis involves changes in the shape of cytoplasmic organelles, chromatin condensation, and oligonucleosomal DNA fragmentation, but without caspase activation (152). Necroptosis is a type of programmed cell death that shares more features with necrosis than apoptosis, and is primarily regulated by receptor-interacting protein kinase-3 (RIPK3) and mixed lineage kinase domain-like protein (MLKL) (153, 154). When apoptosis is blocked, for example, due to bcl-2 family protein dysfunction, aging or apoptotic neutrophils may undergo cell clearance via necroptosis (155).

Although necrosis and necroptosis are typically independent of the caspase cascade, ROS and Ca^2+^ ions are key participants that do not directly relate to external stimuli in these two processes. During necrosis, excessive Ca^2+^ inflow causes mitochondrial calcium overload, and ROS destroys lipid, protein, and DNA structures, which jointly lead to disordered ionic equilibrium and mitochondrial dysfunction. In necrosis, unlike apoptosis, mitochondria usually swell, which greatly impairs OXPHOS and ATP generation. Consequently, apoptosis is blocked due to deficient release of apoptotic proteins from the mitochondria under remote energy supply conditions (156). In necroptosis, ROS generation largely depends on mitochondria, using a mechanism involving mitochondrial permeability transition related to cyclophilin D, rather than Bax or Bak (155). However, necroptosis can be triggered normally in cells with a low density of mitochondria, such as neutrophils (157).

### 3.4 Autophagy

Autophagy is an important cellular process that maintains metabolic equilibrium when energy or nutrients are deficient. In neutrophils, autophagy is indispensable for immune response regulation and pathogen elimination, as neutrophils are often present in environments lacking sufficient oxygen or energy sources. Autophagy can also be responsible for removal of harmful substances, such as damaged mitochondria and protein aggregates in the cytosol, a process some researchers refer to as selective autophagy (158). In addition to selective autophagy, macroautophagy and microautophagy are processes triggered by distinct pathways that result in similar cellular consequences (159).

Autophagy is a form of cell death used to compensate for energy deficiency and inhibits other cell death processes, such as apoptosis or NETosis. For example, dysfunction of PD-L1, which promotes neutrophil autophagy, results in failure of NET formation and release (160). In cases where NETosis is activated by PMA stimulation and energy substrates, such as glucose or glutamine, are relatively restricted, autophagy can be initiated to provide energy for NET release and inhibit apoptosis (161). Under stressful situations, for example, limited glucose, selective autophagy can degrade specific proteins via lysosomes for nutrient and energy supplementation. This process, regulated by metabolic status, is important for maintaining metabolic balance (162). However, this type of autophagy can also consume excessive metabolic proteins, such as hexokinase 2 (HK2), impairing normal cellular metabolism and triggering unexpected cell death (163). Dysregulated selective autophagy primarily affects glucose and lipid metabolism, while its specific effects on neutrophils and neutrophilic metabolism remain elusive (164).

Autophagy is an essential source of FAs for neutrophils. As outlined earlier, autophagy mediates lipolysis to generate FAs for mitochondrial respiration, which provides energy for neutrophil differentiation in the bone marrow autophagy mediates lipolysis to degrade lipid and generate FAs for mitochondrial respiration, which provides energy for neutrophil differentiation in the bone marrow through FAs oxidation (78). Autophagy deficiency inhibits neutrophil degranulation by decreasing ROS production from fatty acid-mediated NAPDH oxidase activity (165). FAs or lipids can also regulate neutrophil autophagy (166, 167). He et al. revealed the role of β-hydroxybutyrate (BHB) in regulating neutrophil autophagy in cows (167). Treatment with BHB facilitates neutrophil adhesion by preventing the degradation of adherent molecules, such as CD11a, CD11b, and CD18, rather than increasing their expression levels. BHB also increases the mRNA abundance and production of the pro-inflammatory factors, IL-1β, IL-6, and TNF in bovine neutrophils, collectively inhibiting neutrophil autophagy (167).

### 3.5 Pyroptosis

Pyroptosis is a newly discovered type of cell death that primarily relies on the activity of caspase-1 and is distinct from other forms of cell death, such as apoptosis. Neutrophil pyroptosis primarily involves processing of specific inflammatory cytokines via the inflammasome to modulate an appropriate immune response, and is usually initiated by intracellular bacterial infections; for example, *Salmonella* carrying *Salmonella* pathogenicity island 1 type III secretion systems (168, 169). *Salmonella* infection boosts assembly and function of the inflammasome, a protein complex that modifies precursors of the inflammatory cytokines, IL-1β and IL-18, driving pyroptosis (168). Activated NLRP3 and NLRC4 inflammasomes promote cleavage of the protein, gasdermin D (GSDMD), and its functional N-terminal proteolytic fragment induces neutrophils to undergo pyroptosis and produce IL-1β (170). In addition to killing intracellular pathogens, pyroptosis is involved in neutrophil secretion of IL-1β and IL-18. Further, in some situations, such as during acute *Salmonella* infection, neutrophils can escape cell death and maintain IL-1β production by activating the NLRC4 inflammasome (171).

Canonical inflammasome activation by *Salmonella* infection also facilitates NETosis and triggers pyroptosis through the NLRP1/3-GSDMD axis (172, 173). NOX2/gp41 deficiency does not block, but rather fosters, compensatory NET release, while GSDMD is unnecessary for PMA-induced NETosis, which largely relies on NAPDH oxidase activity (172). Although mROS is an established NLRP3 inflammasome activator, insufficient neutrophil mitochondrial function limits ROS activity as a powerful regulator of neutrophilic pyroptosis (174, 175). Circulating mitochondrial DNA can act as an effector to activate the canonical inflammasome, and is released on stimulation by calcium influx, along with mROS or other pyroptosis-inducing factors (176, 177). While mitochondria have diverse roles in regulating pyroptosis, there is limited evidence of their significance in neutrophils, which contain low numbers of mitochondria.

In dendritic cells, promotion of pyroptosis involves switching of the major metabolic pathway from OXPHOS to glycolysis, similar to the neutrophilic maturation process (178). Along with other chemical and biological inhibitors of hexokinase, N-acetylglucosamine, a component of the bacterial cell wall, mediates the relocalization of hexokinase from the surface of mitochondria to the cytosol, driving the NLRP3 inflammasome pathway and pyroptosis, independent of mROS and mitochondrial DNA (179).

Upregulated fatty acid supply or synthesis can facilitate inflammation through inflammasome activation (180). In contrast, limited fatty acid availability leads to reduced ROS production and increased autophagy, due to the anti-inflammatory effect of AMP-activated protein kinase, thereby inhibiting other forms of cell death and the inflammatory response (181). However, when glucose is scarce, FAO is abnormally activated to produce sufficient energy. During this process, ketone bodies such as BHB are produced, which can inhibit NLRP3 inflammasome activation through various pathways (182). Butyrate, a short-chain fatty acid with structural similarity to BHB, also has an anti-inflammatory effect by silencing the inflammasome (182). Indeed, short-chain FAs produced by gut microbiota, which are engaged in lipid metabolism in most cell types, have shown remarkable anti-inflammatory effects through various mechanisms, including control of the inflammasome and pyroptosis (183).

### 3.6 Ferroptosis

Ferroptosis is an emerging form of cell death, with some features of necrosis, that has become an increasing focus of attention since it was first described in 2012 (184). Peroxidation of phospholipids mediated by intracellular iron accumulation is the most important characteristic of ferroptosis, which is regulated by a series of cellular and molecular mechanisms. While the roles and mechanisms of ferroptosis in cancer and ischemic diseases have been widely studied, its impact on neutrophil function is poorly understood (185). Nevertheless, several studies have explored the impact of neutrophil ferroptosis on modulating the tumor microenvironment, revealing that it can have both anti- and pro-tumor effects, depending on the context (14, 19, 186).

Lipid metabolism is the most pivotal metabolic pathway involved in ferroptosis across various cell types. Accumulation of cellular lipid peroxides drives ferroptosis; the peroxides are mainly derived from polyunsaturated FAs (PUFAs), such as arachidonic acid, which make up membrane phospholipids (187). The GSH-dependent lipid hydroperoxidase, GSH peroxidase 4 (GPX4), is a crucial regulator of ferroptosis, that can reduce phospholipid hydroperoxides through GSH oxidation, thereby preventing ferroptosis (188). In addition to lipid hydroperoxidases, several endogenous mechanisms eliminate lipid peroxides or prevent their production. Lipophilic radical trapping antioxidants (RTAs), such as tetrahydrobiopterin or coenzyme Q10, are endogenous reductive products that inhibit ferroptosis (189, 190). Endogenous RTA synthesis is triggered by ferroptosis suppressor protein 1 (FSP1), which is located on the plasma membrane, and FSP1 levels can be modulated by the antioxidant transcription factor, NRF2 (190, 191). During ferroptosis, general lipid metabolism, including PUFA metabolism, MUFA metabolism, and phospholipid synthesis and remodeling, is reprogrammed (192).

Although lipid oxidation is a central feature of neutrophilic ferroptosis, its metabolic characteristics, including glucose and glutamine metabolism, have only been explored in a limited number of studies. Glutamine metabolism is critical in neutrophils, and GSH serves as a major antioxidant, levels of which are closely related to cellular oxidative state and ROS production. Gao et al. and Xiao et al. reported that, in cancer cells, ferroptosis is controlled by glutamine metabolism and supply, as well as being regulated by cellular ROS levels (193, 194). Glutamate transporters, such as SLC7A11 and SLC1A5, modulate ferroptosis initiation by adjusting glutamine and GSH levels (195).

Glucose starvation limits neutrophil ROS production, suggesting that ferroptosis is proportionately linked to glucose metabolism. Moreover, energy depletion caused by glucose starvation activates the AMPK pathway, which blocks ferroptosis (196). As an energy-sensing kinase, AMPK is activated by energy insufficiency to inhibit the synthesis of polyunsaturated FAs that are indispensable for ferroptosis (197, 198). However, it remains unclear whether glycolysis or the PPP is the main glucose metabolic pathway during ferroptosis.

Similar to pyroptosis, ferroptosis is regulated by mitochondrial function, as mitochondria contribute substantially to glutamine metabolism, ROS synthesis, and lipid peroxide accumulation (199). although the role of mitochondria in neutrophil ferroptosis is elusive, due to continuous changes in mitochondrial activity and density in these cells.

## 4 Conclusion and future

Studying the metabolic features of immune cells is essential to understanding immune system function and regulation. In recent years, the metabonomics of innate immune cells, particularly neutrophils, has become the focus of increasing research, leading to elucidation of complex characteristics of the innate immune system. Although most innate immune cell types rely heavily on glucose metabolism, neutrophils, natural killer cells, and M2 macrophages predominantly utilize glycolysis, with almost no OXPHOS or respiratory electron transport chain activity (33, 200, 201). On neutrophil activation, the PPP is promoted, alongside glycolysis, to supply NADPH for antibacterial activity. Mitochondria are present at a low density in neutrophils and have altered function, barely participating in neutrophil energy metabolism; hence, the effects of mitochondria regulation on neutrophils are controversial (202). Lipid metabolism generally contributes to neutrophil differentiation and maturation, then decreases with increasing mitochondrial activity and OXPHOS. FAO provides energy for immature neutrophils, while lipid metabolism also contributes to membrane assembly and signal transduction. Glutamine has a broad-spectrum protective effect on the immune system and supplies additional NADPH to neutrophils, thus helping to maintain redox homeostasis. The production and elimination of ROS by different metabolic processes play an essential role in neutrophil function and death. E3 ubiquitin ligase TRIM29 is expressed in neutrophils and can modulate the PERK-mediated ER stress response to effect ROS production (203, 204).TRIM29 and ER stress response may be equipped with the ability to influence neutrophil cell fate, which is to be discussed. As mentioned above, akt significantly determines neutrophil to enter apoptosis or NETosis and PI3K/AKT pathway can be regulated by various metabolic processes (205). Poly(ADP-ribose) polymerase 9 (PARP9) is able to activate PI3K/AKT pathway in various immune cells and is a capable enzyme that bridges neutrophil metabolism and cell death (206). In general, neutrophil activity and metabolism are centered around ATP and ROS synthesis, with glucose and glycolysis as pivotal features, although the contributions of other metabolic pathways are gradually being elucidated.

Cell death does not necessarily indicate loss or impairment of neutrophil function. On the contrary, different types of cell death correspond to distinct immunological signaling pathways and individually modulate the immune response (207). Metabolic reprogramming, initiated by various death processes, is strongly linked to immune response adaptation, and changes in energy demand and metabolite production induced by cell death processes can induce initiation of neutrophil cell death. Apoptosis is the most common form of neutrophil death in inflammatory environments, and is often associated with neutrophil activation. As would be expected, glycolysis and PPP are inhibited as the energy requirements of aged neutrophils diminish. Hypoxia contributes considerably to apoptosis, regardless of the effects of HIF1/2 on transcription, although ROS appears to be a double-edged sword in the context of neutrophil apoptosis. NETosis, the formation and release of NETs, is crucial for neutrophil function and can sometimes occur while cell viability is maintained; energy and ROS are necessary for this process, hence, glucose metabolism, including glycolysis and PPP, as well as glutamine metabolism, are enhanced. In some pathological or abnormal conditions, lipid metabolism pathways can activate NETosis, and the detailed mechanisms occurring in different disease models warrant further investigation. Necrosis and necroptosis function in a ROS-dependent manner, although their specific roles in neutrophils remain unknown. Autophagy generally occurs during neutrophil differentiation, and helps maintain homeostasis of energy and materials; hence, dysfunctional autophagy usually leads to an impaired immune response. Autophagy is both a regulator of and regulated by lipid metabolism, as well as other metabolic pathways, and its role in the neutrophil life cycle, beyond its early stages, remains unknown. Pyroptosis is an essential type of immunological cell death, that aids immune cytokine secretion, alongside the inflammasome. Inflammasome assembly requires the activity of NOX and ROS, while the role of mROS in neutrophils is poorly understood. In neutrophils, pyroptosis drives a unique type of NETosis, distinct from the PMA-induced mechanism, the metabolic features which, including the potential key role of glycolysis, warrant further research. Ferroptosis, which is closely related to lipid metabolism reprogramming, is a current focus in neutrophil research; however, its specific metabolic pathways and alterations, particularly concerning various types of metabolism, require further investigation.

Complicated relationship between metabolism and cell death in neutrophil may reveal that targeting metabolism reprogramming to modulate abnormal cell death is an effective therapy strategy to treat correlative diseases. For GSD-1b patients poorly respond to regular treatment, somatic gene therapy is becoming an available choice. Both adenovirus and adeno-associated virus can carry vectors that express human G6PT. They have shown encouraging ability to correct metabolism impairment, over-activated apoptosis and neutropenia as they are infused into G6pt^−/−^ mice (208, 209). Excessive NETosis have been verified as an essential factor that causes various diseases like sepsis, rheumatic disease and inflammatory bowel disease thus there are a large amount of therapeutics trying to treat these diseases by inhibiting NETosis (210). ROS scavengers like N‐acetylcysteine and Methotrexate, which inhibit NET formation by reducing ROS formation, have shown remarkable therapy benefits in some clinical or preclinical trials (210, 211). As mentioned in chapter 3.2, lipid metabolism bypass activation can also be a reason for aberrant NET formation and it may be a new therapeutic target for cardiovascular and coagulation disorder. ROS is undoubtedly one of the most important metabolites that influence the cell fate as it takes part in almost all the cell death forms in neutrophils. Direct or indirect modulation of ROS production and elimination is an important method to regulate neutrophil cell function and death, then help improve the treatment for diseases. Besides, more relevance of neutrophil metabolism to cell death is still to be explored and there will be more possibilities that cell fate of neutrophil can be modulated by influencing cell death in more diseases.

## References

[1] Hedrick CC Malanchi I. Neutrophils in cancer: heterogeneous and multifaceted. Nat Rev Immunol. (2022) 22:173–87. doi: 10.1038/s41577-021-00571-6 34230649

[2] Borregaard N. Neutrophils, from marrow to microbes. Immunity. (2010) 33:657–70. doi: 10.1016/j.immuni.2010.11.011 21094463

[3] Bugl S Wirths S Müller MR Radsak MP Kopp H-G. Current insights into neutrophil homeostasis. Ann N Y Acad Sci. (2012) 1266:171–8. doi: 10.1111/j.1749-6632.2012.06607.x 22901268

[4] Mehta HM Corey SJ. G-CSF, the guardian of granulopoiesis. Semin Immunol. (2021) 54:101515. doi: 10.1016/j.smim.2021.101515 34772606

[5] Kolaczkowska E Kubes P. Neutrophil recruitment and function in health and inflammation. Nat Rev Immunol. (2013) 13:159–75. doi: 10.1038/nri3399 23435331

[6] Basu S Hodgson G Katz M Dunn AR. Evaluation of role of G-CSF in the production, survival, and release of neutrophils from bone marrow into circulation. Blood. (2002) 100:854–61. doi: 10.1182/blood.V100.3.854 12130495

[7] Galli SJ Borregaard N Wynn TA. Phenotypic and functional plasticity of cells of innate immunity: macrophages, mast cells and neutrophils. Nat Immunol. (2011) 12:1035–44. doi: 10.1038/ni.2109 PMC3412172 22012443

[8] Gabelloni ML Trevani AS Sabatté J Geffner J. Mechanisms regulating neutrophil survival and cell death. Semin Immunopathol. (2013) 35:423–37. doi: 10.1007/s00281-013-0364-x 23370701

[9] Zhu C-L Wang Y Liu Q Li H-R Yu C-M Li P. Dysregulation of neutrophil death in sepsis. Front Immunol. (2022) 13:963955. doi: 10.3389/fimmu.2022.963955 36059483 PMC9434116

[10] Zhao J Wei K Jiang P Chang C Xu L Xu L. Inflammatory response to regulated cell death in gout and its functional implications. Front Immunol. (2022) 13:888306. doi: 10.3389/fimmu.2022.888306 35464445 PMC9020265

[11] Wiegman CH Li F Ryffel B Togbe D Chung KF. Oxidative stress in ozone-induced chronic lung inflammation and emphysema: A facet of chronic obstructive pulmonary disease. Front Immunol. (2020) 11:1957. doi: 10.3389/fimmu.2020.01957 32983127 PMC7492639

[12] Wigerblad G Kaplan MJ. Neutrophil extracellular traps in systemic autoimmune and autoinflammatory diseases. Nat Rev Immunol. (2023) 23:274–88. doi: 10.1038/s41577-022-00787-0 PMC9579530 36257987

[13] Wang J-F Wang Y-P Xie J Zhao Z-Z Gupta S Guo Y. Upregulated PD-L1 delays human neutrophil apoptosis and promotes lung injury in an experimental mouse model of sepsis. Blood. (2021) 138:806–10. doi: 10.1182/blood.2020009417 34473230

[14] Zhao Y Liu Z Liu G Zhang Y Liu S Gan D. Neutrophils resist ferroptosis and promote breast cancer metastasis through aconitate decarboxylase 1. Cell Metab. (2023) 35. doi: 10.1016/j.cmet.2023.09.004 PMC10558089 37793345

[15] Homburg CH Roos D. Apoptosis of neutrophils. Curr Opin Hematol. (1996) 3:94–9. doi: 10.1097/00062752-199603010-00014 9372057

[16] Dejas L Santoni K Meunier E Lamkanfi M. Regulated cell death in neutrophils: From apoptosis to NETosis and pyroptosis. Semin Immunol. (2023) 70:101849. doi: 10.1016/j.smim.2023.101849 37939552 PMC10753288

[17] Pérez-Figueroa E Álvarez-Carrasco P Ortega E Maldonado-Bernal C. Neutrophils: many ways to die. Front Immunol. (2021) 12:631821. doi: 10.3389/fimmu.2021.631821 33746968 PMC7969520

[18] Gao X Zhang W Zhang N Yu Q Su J Wang K. Multiple death pathways of neutrophils regulate alveolar macrophage proliferation. Cells. (2022) 11. doi: 10.3390/cells11223633 PMC9688429 36429062

[19] Kim R Hashimoto A Markosyan N Tyurin VA Tyurina YY Kar G. Ferroptosis of tumour neutrophils causes immune suppression in cancer. Nature. (2022) 612:338–46. doi: 10.1038/s41586-022-05443-0 PMC9875862 36385526

[20] Yipp BG Kubes P. NETosis: how vital is it? Blood. (2013) 122:2784–94. 10.1182/blood-2013-04-457671 24009232

[21] Geering B Simon HU. Peculiarities of cell death mechanisms in neutrophils. Cell Death Differ. (2011) 18:1457–69. doi: 10.1038/cdd.2011.75 PMC3178425 21637292

[22] Lawrence SM Corriden R Nizet V. How neutrophils meet their end. Trends Immunol. (2020) 41:531–44. doi: 10.1016/j.it.2020.03.008 32303452

[23] Savill JS Wyllie AH Henson JE Walport MJ Henson PM Haslett C. Macrophage phagocytosis of aging neutrophils in inflammation. Programmed cell death in the neutrophil leads to its recognition by macrophages. J Clin Invest. (1989) 83:865–75. doi: 10.1172/JCI113970 PMC303760 2921324

[24] Liew PX Kubes P. The neutrophil’s role during health and disease. Physiol Rev. (2019) 99:1223–48. doi: 10.1152/physrev.00012.2018 30758246

[25] Becker EL. Some interrelations of neutrophil chemotaxis, lysosomal enzyme secretion, and phagocytosis as revealed by synthetic peptides. Am J Pathol. (1976) 85:385–94. PMC2032572 793409

[26] Tamassia N Bianchetto-Aguilera F Arruda-Silva F Gardiman E Gasperini S Calzetti F. Cytokine production by human neutrophils: Revisiting the “dark side of the moon. Eur J Clin Invest. (2018) 48 Suppl 2:e12952. doi: 10.1111/eci.2018.48.issue-S2 29772063

[27] de Oliveira S Rosowski EE Huttenlocher A. Neutrophil migration in infection and wound repair: going forward in reverse. Nat Rev Immunol. (2016) 16:378–91. doi: 10.1038/nri.2016.49 PMC5367630 27231052

[28] Giese MA Hind LE Huttenlocher A. Neutrophil plasticity in the tumor microenvironment. Blood. (2019) 133:2159–67. doi: 10.1182/blood-2018-11-844548 PMC6524564 30898857

[29] Jaillon S Ponzetta A Di Mitri D Santoni A Bonecchi R Mantovani A. Neutrophil diversity and plasticity in tumour progression and therapy. Nat Rev Cancer. (2020) 20:485–503. doi: 10.1038/s41568-020-0281-y 32694624

[30] Gonzalez H Hagerling C Werb Z. Roles of the immune system in cancer: from tumor initiation to metastatic progression. Genes Dev. (2018) 32:1267–84. doi: 10.1101/gad.314617.118 PMC6169832 30275043

[31] Plas DR Thompson CB. Cell metabolism in the regulation of programmed cell death. Trends Endocrinol Metab. (2002) 13:75–8. doi: 10.1016/S1043-2760(01)00528-8 11854022

[32] Beck WS Valentine WN. The aerobic carbohydrate metabolism of leukocytes in health and leukemia. I. Glycolysis and respiration. Cancer Res. (1952) 12:818–22. 12998046

[33] Borregaard N Herlin T. Energy metabolism of human neutrophils during phagocytosis. J Clin Invest. (1982) 70:550–7. doi: 10.1172/JCI110647 PMC370256 7107894

[34] Toller-Kawahisa JE O’Neill LAJ. How neutrophil metabolism affects bacterial killing. Open Biol. (2022) 12:220248. doi: 10.1098/rsob.220248 36416011 PMC9682436

[35] Dan Dunn J Alvarez LA Zhang X Soldati T. Reactive oxygen species and mitochondria: A nexus of cellular homeostasis. Redox Biol. (2015) 6:472–85. doi: 10.1016/j.redox.2015.09.005 PMC4596921 26432659

[36] Jiang J Tu H Li P. Lipid metabolism and neutrophil function. Cell Immunol. (2022) 377:104546. doi: 10.1016/j.cellimm.2022.104546 35688009

[37] Lagranha CJ Levada-Pires AC Sellitti DF Procopio J Curi R Pithon-Curi TC. The effect of glutamine supplementation and physical exercise on neutrophil function. Amino Acids. (2008) 34:337–46. doi: 10.1007/s00726-007-0560-x 17928941

[38] Vorobjeva NV Chernyak BV. NETosis: molecular mechanisms, role in physiology and pathology. Biochem (Mosc). (2020) 85:1178–90. doi: 10.1134/S0006297920100065 PMC7590568 33202203

[39] Willson JA Arienti S Sadiku P Reyes L Coelho P Morrison T. Neutrophil HIF-1α stabilization is augmented by mitochondrial ROS produced via the glycerol 3-phosphate shuttle. Blood. (2022) 139:281–6. doi: 10.1182/blood.2021011010 PMC8832465 34411229

[40] Simon HU Haj-Yehia A Levi-Schaffer F. Role of reactive oxygen species (ROS) in apoptosis induction. Apoptosis. (2000) 5:415–8. doi: 10.1023/A:1009616228304 11256882

[41] Morris G Gevezova M Sarafian V Maes M. Redox regulation of the immune response. Cell Mol Immunol. (2022) 19:1079–101. doi: 10.1038/s41423-022-00902-0 PMC9508259 36056148

[42] Michaeloudes C Bhavsar PK Mumby S Xu B Hui CKM Chung KF. Role of metabolic reprogramming in pulmonary innate immunity and its impact on lung diseases. J Innate Immun. (2020) 12:31–46. doi: 10.1159/000504344 31786568 PMC6959099

[43] Shafqat A Khan JA Alkachem AY Sabur H Alkattan K Yaqinuddin A. How neutrophils shape the immune response: reassessing their multifaceted role in health and disease. Int J Mol Sci 24. (2023). doi: 10.3390/ijms242417583 PMC10744338 38139412

[44] Selvaraj RJ Sbarra AJ. Relationship of glycolytic and oxidative metabolism to particle entry and destruction in phagocytosing cells. Nature. (1966) 211:1272–6. doi: 10.1038/2111272a0 5969807

[45] Sbarra AJ Karnovsky ML. The biochemical basis of phagocytosis. I. Metabolic changes during the ingestion of particles by polymorphonuclear leukocytes. J Biol Chem. (1959) 234:1355–62. doi: 10.1016/S0021-9258(18)70011-2 13654378

[46] van Raam BJ Sluiter W de Wit E Roos D Verhoeven AJ Kuijpers TW. Mitochondrial membrane potential in human neutrophils is maintained by complex III activity in the absence of supercomplex organisation. PloS One. (2008) 3:e2013. 18431494 10.1371/journal.pone.0002013 PMC2295260

[47] Gould GW Holman GD. The glucose transporter family: structure, function and tissue-specific expression. Biochem J. (1993) 295:329–41. doi: 10.1042/bj2950329 PMC1134886 8240230

[48] Maratou E Dimitriadis G Kollias A Boutati E Lambadiari V Mitrou P. Glucose transporter expression on the plasma membrane of resting and activated white blood cells. Eur J Clin Invest. (2007) 37:282–90. doi: 10.1111/j.1365-2362.2007.01786.x 17373964

[49] Maianski NA Geissler J Srinivasula SM Alnemri ES Roos D Kuijpers TW. Functional characterization of mitochondria in neutrophils: a role restricted to apoptosis. Cell Death Differ. (2004) 11:143–53. doi: 10.1038/sj.cdd.4401320 14576767

[50] Kierans SJ Taylor CT. Regulation of glycolysis by the hypoxia-inducible factor (HIF): implications for cellular physiology. J Physiol. (2021) 599:23–37. doi: 10.1113/tjp.v599.1 33006160

[51] Injarabian L Devin A Ransac S Marteyn BS. Neutrophil Metabolic Shift during their Lifecycle: Impact on their Survival and Activation. Int J Mol Sci. (2019) 21. doi: 10.3390/ijms21010287 PMC6981538 31906243

[52] Sadiku P Willson JA Ryan EM Sammut D Coelho P Watts ER. Neutrophils fuel effective immune responses through gluconeogenesis and glycogenesis. Cell Metab. (2021) 33. 10.1016/j.cmet.2021.03.018 PMC8102058 33951466

[53] Zhang H Ma J Tang K Huang B. Beyond energy storage: roles of glycogen metabolism in health and disease. FEBS J. (2021) 288:3772–83. doi: 10.1111/febs.v288.12 33249748

[54] Kumar S Dikshit M. Metabolic insight of neutrophils in health and disease. Front Immunol. (2019) 10:2099. doi: 10.3389/fimmu.2019.02099 31616403 PMC6764236

[55] Sadiku P Willson JA Dickinson RS Murphy F Harris AJ Lewis A. Prolyl hydroxylase 2 inactivation enhances glycogen storage and promotes excessive neutrophilic responses. J Clin Invest. (2017) 127:3407–20. doi: 10.1172/JCI90848 PMC5669581 28805660

[56] Chou JY Jun HS Mansfield BC. Neutropenia in type Ib glycogen storage disease. Curr Opin Hematol. (2010) 17:36–42. doi: 10.1097/MOH.0b013e328331df85 19741523 PMC3099242

[57] Chou JY Jun HS Mansfield BC. Glycogen storage disease type I and G6Pase-β deficiency: etiology and therapy. Nat Rev Endocrinol. (2010) 6:676–88. doi: 10.1038/nrendo.2010.189 PMC4178929 20975743

[58] Jun HS Weinstein DA Lee YM Mansfield BC Chou JY. Molecular mechanisms of neutrophil dysfunction in glycogen storage disease type Ib. Blood. (2014) 123:2843–53. doi: 10.1182/blood-2013-05-502435 PMC4007611 24565827

[59] TeSlaa T Ralser M Fan J Rabinowitz JD. The pentose phosphate pathway in health and disease. Nat Metab. (2023) 5:1275–89. doi: 10.1038/s42255-023-00863-2 PMC11251397 37612403

[60] Hostetler KY Landau BR. Estimation of the pentose cycle contribution to glucose metabolism in tissue in *vivo* . Biochemistry. (1967) 6:2961–4. doi: 10.1021/bi00862a001 6056970

[61] Stanton RC. Glucose-6-phosphate dehydrogenase, NADPH, and cell survival. IUBMB Life. (2012) 64:362–9. doi: 10.1002/iub.v64.5 PMC3325335 22431005

[62] Stincone A Prigione A Cramer T Wamelink MMC Campbell K Cheung E. The return of metabolism: biochemistry and physiology of the pentose phosphate pathway. Biol Rev Camb Philos Soc. (2015) 90:927–63. doi: 10.1111/brv.2015.90.issue-3 PMC4470864 25243985

[63] Britt EC Lika J Giese MA Schoen TJ Seim GL Huang Z. Switching to the cyclic pentose phosphate pathway powers the oxidative burst in activated neutrophils. Nat Metab. (2022) 4:389–403. doi: 10.1038/s42255-022-00550-8 35347316 PMC8964420

[64] Siler U Romao S Tejera E Pastukhov O Kuzmenko E Valencia RG. Severe glucose-6-phosphate dehydrogenase deficiency leads to susceptibility to infection and absent NETosis. J Allergy Clin Immunol. (2017) 139. doi: 10.1016/j.jaci.2016.04.041 27458052

[65] Brinkmann V Reichard U Goosmann C Fauler B Uhlemann Y Weiss DS. Neutrophil extracellular traps kill bacteria. Science. (2004) 303:1532–5. doi: 10.1126/science.1092385 15001782

[66] Hirsch JG Fedorko ME. Ultrastructure of human leukocytes after simultaneous fixation with glutaraldehyde and osmium tetroxide and “postfixation” in uranyl acetate. J Cell Biol. (1968) 38:615–27. doi: 10.1083/jcb.38.3.615 PMC2108377 4874495

[67] Edwards SW Hallett MB Campbell AK. Oxygen-radical production during inflammation may be limited by oxygen concentration. Biochem J. (1984) 217:851–4. doi: 10.1042/bj2170851 PMC1153291 6712601

[68] Chacko BK Kramer PA Ravi S Johnson MS Hardy RW Ballinger SW. Methods for defining distinct bioenergetic profiles in platelets, lymphocytes, monocytes, and neutrophils, and the oxidative burst from human blood. Lab Invest. (2013) 93:690–700. doi: 10.1038/labinvest.2013.53 23528848 PMC3674307

[69] Fossati G Moulding DA Spiller DG Moots RJ White MRH Edwards SW. The mitochondrial network of human neutrophils: role in chemotaxis, phagocytosis, respiratory burst activation, and commitment to apoptosis. J Immunol. (2003) 170:1964–72. doi: 10.4049/jimmunol.170.4.1964 12574365

[70] Esposito LA Melov S Panov A Cottrell BA Wallace DC. Mitochondrial disease in mouse results in increased oxidative stress. Proc Natl Acad Sci U.S.A. (1999) 96:4820–5. 10.1073/pnas.96.9.4820 PMC21775 10220377

[71] Bao Y Ledderose C Seier T Graf AF Brix B Chong E. Mitochondria regulate neutrophil activation by generating ATP for autocrine purinergic signaling. J Biol Chem. (2014) 289:26794–803. doi: 10.1074/jbc.M114.572495 PMC4175322 25104353

[72] Vorobjeva N Prikhodko A Galkin I Pletjushkina O Zinovkin R Sud’ina G. Mitochondrial reactive oxygen species are involved in chemoattractant-induced oxidative burst and degranulation of human neutrophils in *vitro* . Eur J Cell Biol. (2017) 96:254–65. doi: 10.1016/j.ejcb.2017.03.003 28325500

[73] Cao Z Zhao M Sun H Hu L Chen Y Fan Z. Roles of mitochondria in neutrophils. Front Immunol. (2022) 13:934444. doi: 10.3389/fimmu.2022.934444 36081497 PMC9447286

[74] Kagawa Y Cha SH Hasegawa K Hamamoto T Endo H. Regulation of energy metabolism in human cells in aging and diabetes: FoF(1), mtDNA, UCP, and ROS. Biochem Biophys Res Commun. (1999) 266:662–76. doi: 10.1006/bbrc.1999.1884 10603304

[75] Letts JA Sazanov LA. Clarifying the supercomplex: the higher-order organization of the mitochondrial electron transport chain. Nat Struct Mol Biol. (2017) 24:800–8. doi: 10.1038/nsmb.3460 28981073

[76] Cunningham CC DeChatelet LR Spach PI Parce JW Thomas MJ Lees CJ. Identification and quantitation of electron-transport components in human polymorphonuclear neutrophils. Biochim Biophys Acta. (1982) 682:430–5. doi: 10.1016/0005-2728(82)90057-3 6295472

[77] Dunham-Snary KJ Surewaard BG Mewburn JD Bentley RE Martin AY Jones O. Mitochondria in human neutrophils mediate killing of Staphylococcus aureus. Redox Biol. (2022) 49:102225. doi: 10.1016/j.redox.2021.102225 34959099 PMC8758915

[78] Riffelmacher T Clarke A Richter FC Stranks A Pandey S Danielli S. Autophagy-dependent generation of free fatty acids is critical for normal neutrophil differentiation. Immunity. (2017) 47. doi: 10.1016/j.immuni.2017.08.005 PMC5610174 28916263

[79] Rice CM Davies LC Subleski JJ Maio N Gonzalez-Cotto M Andrews C. Tumour-elicited neutrophils engage mitochondrial metabolism to circumvent nutrient limitations and maintain immune suppression. Nat Commun. (2018) 9:5099. doi: 10.1038/s41467-018-07505-2 30504842 PMC6269473

[80] Yasugi E Horiuchi A Uemura I Okuma E Nakatsu M Saeki K. Peroxisome proliferator-activated receptor gamma ligands stimulate myeloid differentiation and lipogenensis in human leukemia NB4 cells. Dev Growth Differ. (2006) 48:177–88. doi: 10.1111/j.1440-169X.2006.00855.x 16573735

[81] Inazawa Y Nakatsu M Yasugi E Saeki K Yuo A. Lipid droplet formation in human myeloid NB4 cells stimulated by all trans retinoic acid and granulocyte colony-stimulating factor: possible involvement of peroxisome proliferator-activated receptor gamma. Cell Struct Funct. (2003) 28:487–93. doi: 10.1247/csf.28.487 14745140

[82] Monson EA Crosse KM Das M Helbig KJ. Lipid droplet density alters the early innate immune response to viral infection. PloS One. (2018) 13:e0190597. doi: 10.1371/journal.pone.0190597 29293661 PMC5749834

[83] Nose F Yamaguchi T Kato R Aiuchi T Obama T Hara S. Crucial role of perilipin-3 (TIP47) in formation of lipid droplets and PGE2 production in HL-60-derived neutrophils. PloS One. (2013) 8:e71542. doi: 10.1371/journal.pone.0071542 23936516 PMC3731282

[84] Zechner R Madeo F. Cell biology: Another way to get rid of fat. Nature. (2009) 458:1118–9. doi: 10.1038/4581118a 19407787

[85] Bremer J. Carnitine–metabolism and functions. Physiol Rev. (1983) 63:1420–80. doi: 10.1152/physrev.1983.63.4.1420 6361812

[86] Zechner R Zimmermann R Eichmann TO Kohlwein SD Haemmerle G Lass A. and lipolysis in lipid metabolism and signaling. Cell Metab. (2012) 15:279–91. doi: 10.1016/j.cmet.2011.12.018 PMC3314979 22405066

[87] Chandak PG Radovic B Aflaki E Kolb D Buchebner M Fröhlich E. Efficient phagocytosis requires triacylglycerol hydrolysis by adipose triglyceride lipase. J Biol Chem. (2010) 285:20192–201. doi: 10.1074/jbc.M110.107854 PMC2888432 20424161

[88] Vallerie SN Hotamisligil GS. The role of JNK proteins in metabolism. Sci Transl Med. (2010) 2:60rv5. doi: 10.1126/scitranslmed.3001007 21123811

[89] Montaigne D Butruille L Staels B. PPAR control of metabolism and cardiovascular functions. Nat Rev Cardiol. (2021) 18:809–23. doi: 10.1038/s41569-021-00569-6 34127848

[90] Schlager S Goeritzer M Jandl K Frei R Vujic N Kolb D. Adipose triglyceride lipase acts on neutrophil lipid droplets to regulate substrate availability for lipid mediator synthesis. J Leukoc Biol. (2015) 98:837–50. doi: 10.1189/jlb.3A0515-206R PMC4594763 26109679

[91] Fachi JL Sécca C Rodrigues PB de Mato FCP Luccia B Felipe J. Acetate coordinates neutrophil and ILC3 responses against C. difficile through FFAR2. J Exp Med. (2020) 217. 10.1084/jem.20190489 PMC7062529 31876919

[92] Nagatake T Shiogama Y Inoue A Kikuta J Honda T Tiwari P. The 17,18-epoxyeicosatetraenoic acid-G protein-coupled receptor 40 axis ameliorates contact hypersensitivity by inhibiting neutrophil mobility in mice and cynomolgus macaques. J Allergy Clin Immunol. (2018) 142. doi: 10.1016/j.jaci.2017.09.053 29288079

[93] Pithon-Curi TC Trezena AG Tavares-Lima W Curi R. Evidence that glutamine is involved in neutrophil function. Cell Biochem Funct. (2002) 20:81–6. doi: 10.1002/cbf.v20:2 11979501

[94] Garcia C Pithon-Curi TC de Lourdes Firmano M Pires de Melo M Newsholme P Curi R. Effects of adrenaline on glucose and glutamine metabolism and superoxide production by rat neutrophils. Clin Sci (Lond). (1999) 96:549–55. doi: 10.1042/cs0960549 10334960

[95] Ogle CK Ogle JD Mao JX Simon J Noel JG Li BG. Effect of glutamine on phagocytosis and bacterial killing by normal and pediatric burn patient neutrophils. JPEN J Parenter Enteral Nutr. (1994) 18:128–33. doi: 10.1177/0148607194018002128 8201747

[96] Pithon-Curi TC Levada AC Lopes LR Doi SQ Curi R. Glutamine plays a role in superoxide production and the expression of p47phox, p22phox and gp91phox in rat neutrophils. Clin Sci (Lond). (2002) 103:403–8. doi: 10.1042/cs1030403 12241540

[97] Cruzat VF Pantaleão LC Donato J de Bittencourt PIH Tirapegui J. Oral supplementations with free and dipeptide forms of L-glutamine in endotoxemic mice: effects on muscle glutamine-glutathione axis and heat shock proteins. J Nutr Biochem. (2014) 25:345–52. doi: 10.1016/j.jnutbio.2013.11.009 24524905

[98] Cruzat VF Bittencourt A Scomazzon SP Leite JSM de Bittencourt PIH Tirapegui J. Oral free and dipeptide forms of glutamine supplementation attenuate oxidative stress and inflammation induced by endotoxemia. Nutrition. (2014) 30:602–11. doi: 10.1016/j.nut.2013.10.019 24698353

[99] Liu N Ma X Luo X Zhang Y He Y Dai Z. l-glutamine attenuates apoptosis in porcine enterocytes by regulating glutathione-related redox homeostasis. J Nutr. (2018) 148:526–34. doi: 10.1093/jn/nxx062 29659951

[100] Young VR Ajami AM. Glutamine: the emperor or his clothes? J Nutr. (2001) 131. 10.1093/jn/131.9.2449S 11533293

[101] Galley HF. Oxidative stress and mitochondrial dysfunction in sepsis. Br J Anaesth. (2011) 107:57–64. doi: 10.1093/bja/aer093 21596843

[102] Scheel-Toellner D Wang K Assi LK Webb PR Craddock RM Salmon M. Clustering of death receptors in lipid rafts initiates neutrophil spontaneous apoptosis. Biochem Soc Trans. (2004) 32:679–81. doi: 10.1042/BST0320679 15493986

[103] Krysko DV Vanden Berghe T D’Herde K Vandenabeele P. Apoptosis and necrosis: detection, discrimination and phagocytosis. Methods. (2008) 44:205–21. doi: 10.1016/j.ymeth.2007.12.001 18314051

[104] Krysko DV Vanden Berghe T Parthoens E D’Herde K Vandenabeele P. Methods for distinguishing apoptotic from necrotic cells and measuring their clearance. Methods Enzymol. (2008) 442:307–41. doi: 10.1016/S0076-6879(08)01416-X 18662577

[105] Park HH Lo Y-C Lin S-C Wang L Yang JK Wu H. The death domain superfamily in intracellular signaling of apoptosis and inflammation. Annu Rev Immunol. (2007) 25:561–86. doi: 10.1146/annurev.immunol.25.022106.141656 PMC2904440 17201679

[106] Guicciardi ME Gores GJ. Life and death by death receptors. FASEB J. (2009) 23:1625–37. doi: 10.1096/fj.08-111005 PMC2698650 19141537

[107] Kasahara Y Iwai K Yachie A Ohta K Konno A Seki H. Involvement of reactive oxygen intermediates in spontaneous and CD95 (Fas/APO-1)-mediated apoptosis of neutrophils. Blood. (1997) 89:1748–53. doi: 10.1182/blood.V89.5.1748 9057659

[108] Martinou J-C Youle RJ. Mitochondria in apoptosis: Bcl-2 family members and mitochondrial dynamics. Dev Cell. (2011) 21. doi: 10.1016/j.devcel.2011.06.017 PMC3156409 21763611

[109] Galluzzi L Vitale I Abrams JM Alnemri ES Baehrecke EH Blagosklonny MV. Molecular definitions of cell death subroutines: recommendations of the Nomenclature Committee on Cell Death 2012. Cell Death Differ. (2012) 19:107–20. doi: 10.1038/cdd.2011.96 PMC3252826 21760595

[110] Savill J Fadok V Henson P Haslett C. Phagocyte recognition of cells undergoing apoptosis. Immunol Today. (1993) 14:131–6. doi: 10.1016/0167-5699(93)90215-7 8385467

[111] Savill J Haslett C. Granulocyte clearance by apoptosis in the resolution of inflammation. Semin Cell Biol. (1995) 6:385–93. doi: 10.1016/S1043-4682(05)80009-1 8748146

[112] Jun HS Lee YM Song KD Mansfield BC Chou JY. G-CSF improves murine G6PC3-deficient neutrophil function by modulating apoptosis and energy homeostasis. Blood. (2011) 117:3881–92. doi: 10.1182/blood-2010-08-302059 PMC3083300 21292774

[113] Hannah S Mecklenburgh K Rahman I Bellingan GJ Greening A Haslett C. Hypoxia prolongs neutrophil survival in vitro. FEBS Lett. (1995) 372:233–7. doi: 10.1016/0014-5793(95)00986-J 7556675

[114] Walmsley SR Print C Farahi N Peyssonnaux C Johnson RS Cramer T. Hypoxia-induced neutrophil survival is mediated by HIF-1alpha-dependent NF-kappaB activity. J Exp Med. (2005) 201:105–15. doi: 10.1084/jem.20040624 PMC2212759 15630139

[115] Thompson AAR Elks PM Marriott HM Eamsamarng S Higgins KR Lewis A. Hypoxia-inducible factor 2α regulates key neutrophil functions in humans, mice, and zebrafish. Blood. (2014) 123:366–76. doi: 10.1182/blood-2013-05-500207 PMC3894493 24196071

[116] Lu H Lin J Xu C Sun M Zuo K Zhang X. Cyclosporine modulates neutrophil functions via the SIRT6-HIF-1α-glycolysis axis to alleviate severe ulcerative colitis. Clin Transl Med. (2021) 11:e334. doi: 10.1002/ctm2.v11.2 33634990 PMC7882115

[117] Lin Q Cong X Yun Z. Differential hypoxic regulation of hypoxia-inducible factors 1alpha and 2alpha. Mol Cancer Res. (2011) 9:757–65. doi: 10.1158/1541-7786.MCR-11-0053 PMC3117969 21571835

[118] Roth E. Nonnutritive effects of glutamine. J Nutr. (2008) 138:2025S–31S. doi: 10.1093/jn/138.10.2025S 18806119

[119] Cruzat V Macedo Rogero M Noel Keane K Curi R Newsholme P. Glutamine: metabolism and immune function, supplementation and clinical translation. Nutrients. (2018) 10. doi: 10.3390/nu10111564 PMC6266414 30360490

[120] Lagranha CJ Hirabara SM Curi R Pithon-Curi TC. Glutamine supplementation prevents exercise-induced neutrophil apoptosis and reduces p38 MAPK and JNK phosphorylation and p53 and caspase 3 expression. Cell Biochem Funct. (2007) 25:563–9. doi: 10.1002/cbf.v25:5 17542038

[121] Kim M-H Kim H. The roles of glutamine in the intestine and its implication in intestinal diseases. Int J Mol Sci. (2017) 18. doi: 10.3390/ijms18051051 PMC5454963 28498331

[122] Lodhi IJ Wei X Yin L Feng C Adak S Abou-Ezzi G. Peroxisomal lipid synthesis regulates inflammation by sustaining neutrophil membrane phospholipid composition and viability. Cell Metab. (2015) 21:51–64. doi: 10.1016/j.cmet.2014.12.002 25565205 PMC4287274

[123] Gilroy DW Colville-Nash PR McMaster S Sawatzky DA Willoughby DA Lawrence T. Inducible cyclooxygenase-derived 15-deoxy(Delta)12-14PGJ2 brings about acute inflammatory resolution in rat pleurisy by inducing neutrophil and macrophage apoptosis. FASEB J. (2003) 17:2269–71. doi: 10.1096/fj.02-1162fje 14563690

[124] Bishop-Bailey D Hla T. Endothelial cell apoptosis induced by the peroxisome proliferator-activated receptor (PPAR) ligand 15-deoxy-Delta12, 14-prostaglandin J2. J Biol Chem. (1999) 274:17042–8. doi: 10.1074/jbc.274.24.17042 10358055

[125] Clària J. Regulation of cell proliferation and apoptosis by bioactive lipid mediators. Recent Pat Anticancer Drug Discovery. (2006) 1:369–82. doi: 10.2174/157489206778776961 18221047

[126] Moulding DA Quayle JA Hart CA Edwards SW. Mcl-1 expression in human neutrophils: regulation by cytokines and correlation with cell survival. Blood. (1998) 92:2495–502. doi: 10.1182/blood.V92.7.2495 9746790

[127] Takei H Araki A Watanabe H Ichinose A Sendo F. Rapid killing of human neutrophils by the potent activator phorbol 12-myristate 13-acetate (PMA) accompanied by changes different from typical apoptosis or necrosis. J Leukoc Biol. (1996) 59:229–40. doi: 10.1002/jlb.59.2.229 8603995

[128] Fuchs TA Abed U Goosmann C Hurwitz R Schulze I Wahn V. Novel cell death program leads to neutrophil extracellular traps. J Cell Biol. (2007) 176:231–41. doi: 10.1083/jcb.200606027 PMC2063942 17210947

[129] Yousefi S Simon D Stojkov D Karsonova A Karaulov A Simon H-U. *In vivo* evidence for extracellular DNA trap formation. Cell Death Dis. (2020) 11:300. doi: 10.1038/s41419-020-2497-x 32355207 PMC7193637

[130] Galluzzi L Vitale I Aaronson SA Abrams JM Adam D Agostinis P. Molecular mechanisms of cell death: recommendations of the Nomenclature Committee on Cell Death 2018. Cell Death Differ. (2018) 25:486–541. doi: 10.1038/s41418-017-0012-4 29362479 PMC5864239

[131] Thiam HR Wong SL Wagner DD Waterman CM. Cellular mechanisms of NETosis. Annu Rev Cell Dev Biol. (2020) 36:191–218. doi: 10.1146/annurev-cellbio-020520-111016 32663035 PMC8499668

[132] Remijsen Q Vanden Berghe T Wirawan E Asselbergh B Parthoens E Rycke R. Neutrophil extracellular trap cell death requires both autophagy and superoxide generation. Cell Res. (2011) 21:290–304. doi: 10.1038/cr.2010.150 21060338 PMC3193439

[133] Khan MA Farahvash A Douda DN Licht J-C Grasemann H Sweezey N. JNK activation turns on LPS- and gram-negative bacteria-induced NADPH oxidase-dependent suicidal NETosis. Sci Rep. (2017) 7:3409. doi: 10.1038/s41598-017-03257-z 28611461 PMC5469795

[134] Douda DN Yip L Khan MA Grasemann H Palaniyar N. Akt is essential to induce NADPH-dependent NETosis and to switch the neutrophil death to apoptosis. Blood. (2014) 123:597–600. doi: 10.1182/blood-2013-09-526707 24458280

[135] Azzouz D Khan MA Sweezey N Palaniyar N. Two-in-one: UV radiation simultaneously induces apoptosis and NETosis. Cell Death Discovery. (2018) 4:51. doi: 10.1038/s41420-018-0048-3 PMC5919968 29736268

[136] Baker EH Clark N Brennan AL Fisher DA Gyi KM Hodson ME. Hyperglycemia and cystic fibrosis alter respiratory fluid glucose concentrations estimated by breath condensate analysis. J Appl Physiol. (1985) 102:1969–75. doi: 10.1152/japplphysiol.01425.2006 17303703

[137] Pité H Morais-Almeida M Rocha SM. Metabolomics in asthma: where do we stand? Curr Opin Pulm Med. (2018) 24. 10.1097/MCP.0000000000000437 29059088

[138] Rodríguez-Espinosa O Rojas-Espinosa O Moreno-Altamirano MMB López-Villegas EO Sánchez-García FJ. Metabolic requirements for neutrophil extracellular traps formation. Immunology. (2015) 145:213–24. doi: 10.1111/imm.2015.145.issue-2 PMC4427386 25545227

[139] Laval J Touhami J Herzenberg LA Conrad C Taylor N Battini J-L. Metabolic adaptation of neutrophils in cystic fibrosis airways involves distinct shifts in nutrient transporter expression. J Immunol. (2013) 190:6043–50. doi: 10.4049/jimmunol.1201755 23690474

[140] Amara N Cooper MP Voronkova MA Webb BA Lynch EM Kollman JM. Selective activation of PFKL suppresses the phagocytic oxidative burst. Cell. (2021) 184. doi: 10.1016/j.cell.2021.07.004 PMC8802628 34320407

[141] Azevedo EP Rochael NC Guimarães-Costa AB de Souza-Vieira TS Ganilho J Saraiva EM. and phorbol 12-myristate 13-acetate-induced neutrophil extracellular trap (NET) formation. J Biol Chem. (2015) 290:22174–83. doi: 10.1074/jbc.M115.640094 PMC4571968 26198639

[142] Bersenev A Wu C Balcerek J Tong W. Lnk controls mouse hematopoietic stem cell self-renewal and quiescence through direct interactions with JAK2. J Clin Invest. (2008) 118:2832–44. doi: 10.1172/JCI35808 PMC2447929 18618018

[143] Tong W Lodish HF. Lnk inhibits Tpo-mpl signaling and Tpo-mediated megakaryocytopoiesis. J Exp Med. (2004) 200:569–80. doi: 10.1084/jem.20040762 PMC2212736 15337790

[144] Wang W Tang Y Wang Y Tascau L Balcerek J Tong W. LNK/SH2B3 loss of function promotes atherosclerosis and thrombosis. Circ Res. (2016) 119. doi: 10.1161/CIRCRESAHA.116.308955 PMC5016083 27430239

[145] Dou H Kotini A Liu W Fidler T Endo-Umeda K Sun X. Oxidized phospholipids promote NETosis and arterial thrombosis in LNK(SH2B3) deficiency. Circulation. (2021) 144:1940–54. doi: 10.1161/CIRCULATIONAHA.121.056414 PMC8663540 34846914

[146] Wong SL Demers M Martinod K Gallant M Wang Y Goldfine AB. Diabetes primes neutrophils to undergo NETosis, which impairs wound healing. Nat Med. (2015) 21:815–9. doi: 10.1038/nm.3887 PMC4631120 26076037

[147] Shah MS Brownlee M. Molecular and cellular mechanisms of cardiovascular disorders in diabetes. Circ Res. (2016) 118:1808–29. doi: 10.1161/CIRCRESAHA.116.306923 PMC4888901 27230643

[148] Rohrbach AS Slade DJ Thompson PR Mowen KA. Activation of PAD4 in NET formation. Front Immunol. (2012) 3:360. doi: 10.3389/fimmu.2012.00360 23264775 PMC3525017

[149] Fadini GP Menegazzo L Scattolini V Gintoli M Albiero M Avogaro A. A perspective on NETosis in diabetes and cardiometabolic disorders. Nutr Metab Cardiovasc Dis. (2016) 26:1–8. doi: 10.1016/j.numecd.2015.11.008 26719220

[150] Westerterp M Fotakis P Ouimet M Bochem AE Zhang H Molusky MM. Cholesterol efflux pathways suppress inflammasome activation, NETosis, and atherogenesis. Circulation. (2018) 138:898–912. doi: 10.1161/CIRCULATIONAHA.117.032636 29588315 PMC6160368

[151] Yalcinkaya M Fotakis P Liu W Endo-Umeda K Dou H Abramowicz S. Cholesterol accumulation in macrophages drives NETosis in atherosclerotic plaques via IL-1β secretion. Cardiovasc Res. (2023) 119:969–81. doi: 10.1093/cvr/cvac189 PMC10153645 36537208

[152] Golstein P Kroemer G. Cell death by necrosis: towards a molecular definition. Trends Biochem Sci. (2007) 32:37–43. doi: 10.1016/j.tibs.2006.11.001 17141506

[153] He S Wang L Miao L Wang T Du F Zhao L. Receptor interacting protein kinase-3 determines cellular necrotic response to TNF-alpha. Cell. (2009) 137:1100–11. doi: 10.1016/j.cell.2009.05.021 19524512

[154] Wang X He Z Liu H Yousefi S Simon H-U. Neutrophil necroptosis is triggered by ligation of adhesion molecules following GM-CSF priming. J Immunol. (2016) 197:4090–100. doi: 10.4049/jimmunol.1600051 27815445

[155] Christofferson DE Yuan J. Necroptosis as an alternative form of programmed cell death. Curr Opin Cell Biol. (2010) 22:263–8. doi: 10.1016/j.ceb.2009.12.003 PMC2854308 20045303

[156] Vakifahmetoglu-Norberg H Ouchida AT Norberg E. The role of mitochondria in metabolism and cell death. Biochem Biophys Res Commun. (2017) 482:426–31. doi: 10.1016/j.bbrc.2016.11.088 28212726

[157] Tait SWG Oberst A Quarato G Milasta S Haller M Wang R. Widespread mitochondrial depletion via mitophagy does not compromise necroptosis. Cell Rep. (2013) 5:878–85. doi: 10.1016/j.celrep.2013.10.034 PMC4005921 24268776

[158] Dikic I Elazar Z. Mechanism and medical implications of mammalian autophagy. Nat Rev Mol Cell Biol. (2018) 19:349–64. doi: 10.1038/s41580-018-0003-4 29618831

[159] Cuervo AM. Autophagy: many paths to the same end. Mol Cell Biochem. (2004) 263:55–72. doi: 10.1023/B:MCBI.0000041848.57020.57 27520665

[160] Zhu C-L Xie J Zhao Z-Z Li P Liu Q Guo Y. PD-L1 maintains neutrophil extracellular traps release by inhibiting neutrophil autophagy in endotoxin-induced lung injury. Front Immunol. (2022) 13:949217. doi: 10.3389/fimmu.2022.949217 36016930 PMC9396256

[161] Wang S Zheng S Zhang Q Yang Z Yin K Xu S. Atrazine hinders PMA-induced neutrophil extracellular traps in carp via the promotion of apoptosis and inhibition of ROS burst, autophagy and glycolysis. Environ pollut. (2018) 243:282–91. doi: 10.1016/j.envpol.2018.08.070 30193222

[162] Schneider JL Suh Y Cuervo AM. Deficient chaperone-mediated autophagy in liver leads to metabolic dysregulation. Cell Metab. (2014) 20:417–32. doi: 10.1016/j.cmet.2014.06.009 PMC4156578 25043815

[163] Xia H-G Najafov A Geng J Galan-Acosta L Han X Guo Y. Degradation of HK2 by chaperone-mediated autophagy promotes metabolic catastrophe and cell death. J Cell Biol. (2015) 210:705–16. doi: 10.1083/jcb.201503044 PMC4555813 26323688

[164] Tasset I Cuervo AM. Role of chaperone-mediated autophagy in metabolism. FEBS J. (2016) 283:2403–13. doi: 10.1111/febs.2016.283.issue-13 PMC4935551 26854402

[165] Bhattacharya A Wei Q Shin JN Abdel Fattah E Bonilla DL Xiang Q. Autophagy is required for neutrophil-mediated inflammation. Cell Rep. (2015) 12:1731–9. doi: 10.1016/j.celrep.2015.08.019 26344765

[166] Peng Z Zhao C Du X Yang Y Li Y Song Y. Autophagy induced by palmitic acid regulates neutrophil adhesion through the granule-dependent degradation of αMβ2 integrin in dairy cows with fatty liver. Front Immunol. (2021) 12:726829. doi: 10.3389/fimmu.2021.726829 34691032 PMC8529007

[167] He J Wang K Liu M Zeng W Li D Majigsuren Z. [amp]]beta;-hydroxybutyrate enhances bovine neutrophil adhesion by inhibiting autophagy. Front Immunol. (2022) 13:1096813. 36713365 10.3389/fimmu.2022.1096813 PMC9874688

[168] Miao EA Leaf IA Treuting PM Mao DP Dors M Sarkar A. Caspase-1-induced pyroptosis is an innate immune effector mechanism against intracellular bacteria. Nat Immunol. (2010) 11:1136–42. doi: 10.1038/ni.1960 PMC3058225 21057511

[169] Jorgensen I Miao EA. Pyroptotic cell death defends against intracellular pathogens. Immunol Rev. (2015) 265:130–42. doi: 10.1111/imr.2015.265.issue-1 PMC4400865 25879289

[170] W.-t. He H Hu L Chen P Wang X Huang Z Yang Z-H. Gasdermin D is an executor of pyroptosis and required for interleukin-1β secretion. Cell Res. (2015) 25:1285–98. 10.1038/cr.2015.139 PMC4670995 26611636

[171] Chen KW Groß CJ Sotomayor FV Stacey KJ Tschopp J Sweet MJ. The neutrophil NLRC4 inflammasome selectively promotes IL-1β maturation without pyroptosis during acute Salmonella challenge. Cell Rep. (2014) 8:570–82. doi: 10.1016/j.celrep.2014.06.028 25043180

[172] Chauhan D Demon D Vande Walle L Paerewijck O Zecchin A Bosseler L. GSDMD drives canonical inflammasome-induced neutrophil pyroptosis and is dispensable for NETosis. EMBO Rep. (2022) 23:e54277. doi: 10.15252/embr.202154277 35899491 PMC9535806

[173] Cao Y Shi M Liu L Zuo Y Jia H Min X. Inhibition of neutrophil extracellular trap formation attenuates NLRP1-dependent neuronal pyroptosis via STING/IRE1α pathway after traumatic brain injury in mice. Front Immunol. (2023) 14:1125759. doi: 10.3389/fimmu.2023.1125759 37143681 PMC10152368

[174] Swanson KV Deng M Ting JPY. The NLRP3 inflammasome: molecular activation and regulation to therapeutics. Nat Rev Immunol. (2019) 19:477–89. doi: 10.1038/s41577-019-0165-0 PMC7807242 31036962

[175] Zhou R Yazdi AS Menu P Tschopp J. A role for mitochondria in NLRP3 inflammasome activation. Nature. (2011) 469:221–5. doi: 10.1038/nature09663 21124315

[176] Zhang Q Raoof M Chen Y Sumi Y Sursal T Junger W. Circulating mitochondrial DAMPs cause inflammatory responses to injury. Nature. (2010) 464:104–7. doi: 10.1038/nature08780 PMC2843437 20203610

[177] Lemasters JJ Theruvath TP Zhong Z Nieminen A-L. Mitochondrial calcium and the permeability transition in cell death. Biochim Biophys Acta. (2009) 1787:1395–401. doi: 10.1016/j.bbabio.2009.06.009 PMC2730424 19576166

[178] Krawczyk CM Holowka T Sun J Blagih J Amiel E DeBerardinis RJ. Toll-like receptor-induced changes in glycolytic metabolism regulate dendritic cell activation. Blood. (2010) 115:4742–9. doi: 10.1182/blood-2009-10-249540 PMC2890190 20351312

[179] Wolf AJ Reyes CN Liang W Becker C Shimada K Wheeler ML. Hexokinase is an innate immune receptor for the detection of bacterial peptidoglycan. Cell. (2016) 166:624–36. doi: 10.1016/j.cell.2016.05.076 PMC5534359 27374331

[180] Wen H Gris D Lei Y Jha S Zhang L Huang MT-H. Fatty acid-induced NLRP3-ASC inflammasome activation interferes with insulin signaling. Nat Immunol. (2011) 12:408–15. doi: 10.1038/ni.2022 PMC4090391 21478880

[181] Li X-N Song J Zhang L LeMaire SA Hou X Zhang C. Activation of the AMPK-FOXO3 pathway reduces fatty acid-induced increase in intracellular reactive oxygen species by upregulating thioredoxin. Diabetes. (2009) 58:2246–57. doi: 10.2337/db08-1512 PMC2750236 19592618

[182] Youm Y-H Nguyen KY Grant RW Goldberg EL Bodogai M Kim D. The ketone metabolite β-hydroxybutyrate blocks NLRP3 inflammasome-mediated inflammatory disease. Nat Med. (2015) 21:263–9. doi: 10.1038/nm.3804 PMC4352123 25686106

[183] Hughes MM O’Neill LAJ. Metabolic regulation of NLRP3. Immunol Rev. (2018) 281:88–98. doi: 10.1111/imr.2018.281.issue-1 29247992

[184] Dixon SJ Lemberg KM Lamprecht MR Skouta R Zaitsev EM Gleason CE. Ferroptosis: an iron-dependent form of nonapoptotic cell death. Cell. (2012) 149:1060–72. doi: 10.1016/j.cell.2012.03.042 PMC3367386 22632970

[185] Jiang X Stockwell BR Conrad M. Ferroptosis: mechanisms, biology and role in disease. Nat Rev Mol Cell Biol. (2021) 22:266–82. doi: 10.1038/s41580-020-00324-8 PMC8142022 33495651

[186] Yee PP Wei Y Kim S-Y Lu T Chih SY Lawson C. Neutrophil-induced ferroptosis promotes tumor necrosis in glioblastoma progression. Nat Commun. (2020) 11:5424. doi: 10.1038/s41467-020-19193-y 33110073 PMC7591536

[187] Kagan VE Mao G Qu F Angeli JPF Doll S Croix CS. Oxidized arachidonic and adrenic PEs navigate cells to ferroptosis. Nat Chem Biol. (2017) 13:81–90. doi: 10.1038/nchembio.2238 27842066 PMC5506843

[188] Yang WS SriRamaratnam R Welsch ME Shimada K Skouta R Viswanathan VS. Regulation of ferroptotic cancer cell death by GPX4. Cell. (2014) 156:317–31. doi: 10.1016/j.cell.2013.12.010 PMC4076414 24439385

[189] Kraft VAN Bezjian CT Pfeiffer S Ringelstetter L Müller C Zandkarimi F. GTP cyclohydrolase 1/tetrahydrobiopterin counteract ferroptosis through lipid remodeling. ACS Cent Sci. (2020) 6:41–53. doi: 10.1021/acscentsci.9b01063 31989025 PMC6978838

[190] Bersuker K Hendricks JM Li Z Magtanong L Ford B Tang PH. The CoQ oxidoreductase FSP1 acts parallel to GPX4 to inhibit ferroptosis. Nature. (2019) 575:688–92. doi: 10.1038/s41586-019-1705-2 PMC6883167 31634900

[191] Koppula P Lei G Zhang Y Yan Y Mao C Kondiparthi L. A targetable CoQ-FSP1 axis drives ferroptosis- and radiation-resistance in KEAP1 inactive lung cancers. Nat Commun. (2022) 13:2206. doi: 10.1038/s41467-022-29905-1 35459868 PMC9033817

[192] Pope LE Dixon SJ. Regulation of ferroptosis by lipid metabolism. Trends Cell Biol. (2023) 33:1077–87. doi: 10.1016/j.tcb.2023.05.003 PMC10733748 37407304

[193] Xiao Z Deng S Liu H Wang R Liu Y Dai Z. Glutamine deprivation induces ferroptosis in pancreatic cancer cells. Acta Biochim Biophys Sin (Shanghai). (2023) 55:1288–300. doi: 10.3724/abbs.2023029 PMC10449637 36942991

[194] Gao M Monian P Quadri N Ramasamy R Jiang X. Glutaminolysis and transferrin regulate ferroptosis. Mol Cell. (2015) 59:298–308. doi: 10.1016/j.molcel.2015.06.011 26166707 PMC4506736

[195] Koppula P Zhuang L Gan B. Cystine transporter SLC7A11/xCT in cancer: ferroptosis, nutrient dependency, and cancer therapy. Protein Cell. (2021) 12:599–620. doi: 10.1007/s13238-020-00789-5 33000412 PMC8310547

[196] Lee H Zandkarimi F Zhang Y Meena JK Kim J Zhuang L. Energy-stress-mediated AMPK activation inhibits ferroptosis. Nat Cell Biol. (2020) 22:225–34. doi: 10.1038/s41556-020-0461-8 PMC7008777 32029897

[197] Yang WS Kim KJ Gaschler MM Patel M Shchepinov MS Stockwell BR. Peroxidation of polyunsaturated fatty acids by lipoxygenases drives ferroptosis. Proc Natl Acad Sci U.S.A. (2016) 113:E4966–75. doi: 10.1073/pnas.1603244113 PMC5003261 27506793

[198] Li C Dong X Du W Shi X Chen K Zhang W. LKB1-AMPK axis negatively regulates ferroptosis by inhibiting fatty acid synthesis. Signal Transduct Target Ther. (2020) 5:187. doi: 10.1038/s41392-020-00297-2 32883948 PMC7471309

[199] Gao M Yi J Zhu J Minikes AM Monian P Thompson CB. Role of mitochondria in ferroptosis. Mol Cell. (2019) 73. doi: 10.1016/j.molcel.2018.10.042 PMC6338496 30581146

[200] Zhu L Zhao Q Yang T Ding W Zhao Y. Cellular metabolism and macrophage functional polarization. Int Rev Immunol. (2015) 34. doi: 10.3109/08830185.2014.969421 25340307

[201] Gardiner CM. NK cell metabolism. J Leukoc Biol. (2019) 105:1235–42. doi: 10.1002/JLB.MR0718-260R 30676653

[202] Kaplan MJ. Mitochondria shape neutrophils during hypoxia. Blood. (2022) 139:159–60. doi: 10.1182/blood.2021013440 PMC8759528 35024809

[203] Xing J Weng L Yuan B Wang Z Jia L Jin R. Identification of a role for TRIM29 in the control of innate immunity in the respiratory tract. Nat Immunol. (2016) 17:1373–80. doi: 10.1038/ni.3580 PMC5558830 27695001

[204] Wang J Lu W Zhang J Du Y Fang M Zhang A. Loss of TRIM29 mitigates viral myocarditis by attenuating PERK-driven ER stress response in male mice. Nat Commun. (2024) 15:3481. doi: 10.1038/s41467-024-44745-x 38664417 PMC11045800

[205] Fontana F Giannitti G Marchesi S Limonta P. The PI3K/akt pathway and glucose metabolism: A dangerous liaison in cancer. Int J Biol Sci. (2024) 20:3113–25. doi: 10.7150/ijbs.89942 PMC11186371 38904014

[206] Xing J Zhang A Du Y Fang M Minze LJ Liu Y-J. Identification of poly(ADP-ribose) polymerase 9 (PARP9) as a noncanonical sensor for RNA virus in dendritic cells. Nat Commun. (2021) 12:2681. doi: 10.1038/s41467-021-23003-4 33976210 PMC8113569

[207] Tu H Ren H Jiang J Shao C Shi Y Li P. Dying to defend: neutrophil death pathways and their implications in immunity. Adv Sci (Weinh). (2024) 11:e2306457. doi: 10.1002/advs.202306457 38044275 PMC10885667

[208] Yiu WH Pan CJ Allamarvdasht M Kim SY Chou JY. Glucose-6-phosphate transporter gene therapy corrects metabolic and myeloid abnormalities in glycogen storage disease type Ib mice. Gene Ther. (2007) 14:219–26. doi: 10.1038/sj.gt.3302869 PMC2507880 17006547

[209] Yiu WH Pan C-J Mead PA Starost MF Mansfield BC Chou JY. Normoglycemia alone is insufficient to prevent long-term complications of hepatocellular adenoma in glycogen storage disease type Ib mice. J Hepatol. (2009) 51:909–17. doi: 10.1016/j.jhep.2008.11.026 PMC2762018 19376605

[210] Huang J Hong W Wan M Zheng L. Molecular mechanisms and therapeutic target of NETosis in diseases. MedComm (2020). (2022) 3:e162. doi: 10.1002/mco2.v3.3 36000086 PMC9390875

[211] Craver BM Ramanathan G Hoang S Chang X Mendez Luque LF Brooks S. N-acetylcysteine inhibits thrombosis in a murine model of myeloproliferative neoplasm. Blood Adv. (2020) 4:312–21. doi: 10.1182/bloodadvances.2019000967 PMC6988398 31978215

